# PREparedness, REsponse and SySTemic transformation (PRE-RE-SyST): a model for disability-inclusive pandemic responses and systemic disparities reduction derived from a scoping review and thematic analysis

**DOI:** 10.1186/s12939-021-01526-y

**Published:** 2021-09-14

**Authors:** Tiago S. Jesus, Sureshkumar Kamalakannan, Sutanuka Bhattacharjya, Yelena Bogdanova, Juan Carlos Arango-Lasprilla, Jacob Bentley, Michel D. Landry, Christina Papadimitriou, Refugee Empowerment Task Force and International Networking Group of the American Congress of Rehabilitation Medicine

**Affiliations:** 1 Global Health and Tropical Medicine (GHTM) & WHO Collaborating Centre for Health Workforce Policy and Planning, Institute of Hygiene and Tropical Medicine - NOVA University of Lisbon, Lisbon, Portugal; 2 Department of Occupational Therapy, College of Health & Rehabilitation Sciences: Sargent College, Boston University, Boston, MA USA; 3 Public Health Foundation of India (PHFI), South Asia Centre for Disability Inclusive Development and Research (SACDIR), Indian Institute of Public Health – Hyderabad (IIPH-H), Hyderabad, India; 4 Department of Occupational Therapy, Byrdine F. Lewis College of Nursing and Health Professions, Georgia State University, Atlanta, USA; 5 Physical Medicine & Rehabilitation Service, VA Boston Healthcare System, Boston, MA USA; 6 Department of Psychiatry, Boston University School of Medicine, Boston, MA USA; 7 IKERBASQUE. Basque Foundation for Science, Bilbao, Spain; 8 Biocruces Bizkaia Health Research Institute, Barakaldo, Spain; 9 Department of Cell Biology and Histology, University of the Basque Country UPV/EHU, Leioa, Spain; 10 Department of Clinical Psychology Seattle Pacific University, Seattle WA, USA; 11 Department of Physical Medicine & Rehabilitation, Johns Hopkins School of Medicine, Baltimore, USA; 12 Duke Global Health Institute (DGHI), School of Medicine, Duke University, Durham, NC USA; 13 Duke-Margolis Center for Health Policy, Duke University, Durham, NC USA; 14 School of Health Sciences, Departments of Interdisciplinary Health Sciences, and Sociology, Oakland University, Rochester, MI USA

**Keywords:** COVID-19, Health equity, Social determinants of health, Disabled persons, Public health

## Abstract

**Background:**

People with disabilities (PwD) have been facing multiple health, social, and economic disparities during the COVID-19 pandemic, stemming from structural disparities experienced for long time. This paper aims to present the PREparedness, RESponse and SySTemic transformation (PRE-RE-SyST): a model for a disability-inclusive pandemic responses and systematic disparities reduction.

**Methods:**

Scoping review with a thematic analysis was conducted on the literature published up to mid-September 2020, equating to the initial stages of the COVID-19 pandemic. Seven scientific databases and three preprint databases were searched to identify empirical or perspective papers addressing health and socio-economic disparities experienced by PwD as well as reporting actions to address them. Snowballing searches and experts’ consultation were also conducted. Two independent reviewers made eligibility decisions and performed data extractions on any action or recommended action to address disparities. A thematic analysis was then used for the model construction, informed by a systems-thinking approach (i.e., the Iceberg Model).

**Results:**

From 1027 unique references, 84 were included in the final analysis. The PRE-RE-SyST model articulates a four-level strategic action to: 1) Respond to prevent or reduce disability disparities during a pandemic crisis; 2) Prepare ahead for pandemic and other crises responses; 3) Design systems and policies for a structural disability-inclusiveness; and 4) Transform society’s cultural assumptions about disability. ‘Simple rules’ and literature-based examples on how these strategies can be deployed are provided.

**Conclusion:**

The PRE-RE-SyST model articulates main strategies, ‘simple rules’ and possible means whereby public health authorities, policy-makers, and other stakeholders can address disability disparities in pandemic crises, and beyond. Beyond immediate pandemic responses, disability-inclusiveness is needed to develop everyday equity-oriented policies and practices that can transform societies towards greater resiliency, as a whole, to pandemic and other health and social emergencies.

**Supplementary Information:**

The online version contains supplementary material available at 10.1186/s12939-021-01526-y.

## Background

People with disabilities (PwD) refer to people who, at any given point in their lifespan, experience any mobility, intellectual, cognitive, development, or sensorial impairments, which, in interaction with environmental factors, affect their daily functioning and social participation [1–3]. PwD also refer to a minority group, frequently vulnerable to stigma, discrimination, marginalization, and socially-determined disadvantages [4, 5]. For a long time, PwD have been experiencing disparities in healthcare access, healthcare quality, and health outcomes that have been well documented [6–8]. In turn, broader discrimination experienced by PwD have been in part driven by ‘ableism’ or ‘ableist’ perspectives, i.e., societal practices and discourses that enforce normalcy assumptions, and disvalue the lives of PwD [9–11].

Like other minority or socially-disadvantaged populations who have experienced an exacerbation of existing health and social inequalities during the COVID-19 pandemic, [12–15] PwD can also be disproportionally affected by the pandemic [16–19]. Disproportional health impacts include: greater risks of being infected with the COVID-19/SAR-COV-2, especially for PwD living in residential or long-term care institutions, [18, 20] greater risks of having more severe health consequences (e.g. higher death rates) once infected, especially among younger PwD compared to non-disabled counter-parts, [18, 21] and finally greater risks of experiencing unethical disadvantages in having access to life-saving treatments – including healthcare workers’ biased assumptions about disability and rationing guidelines that do not comply with antidiscrimination rights and laws [9, 10, 18, 22–25].

Beyond higher health risks from a COVID-infection, PwD can experience disproportionate impacts of lockdown-related measures to control the pandemic [19]. These include limited access to key health and rehabilitation services PwD often rely on, disruption in community support networks that help PwD to live independently in the community, and interruption of special education and therapeutic services that provide individualized support and life structure to children with disabilities, among other disproportionate impacts [4, 19, 26–28]. Overall, unintended effects of lockdown-related measures affect the health, social participation, and socio-economic well-being of PwD and their informal caregivers [19].

Overall, we argue that public health and policy responses to the COVID-19 pandemic, as well as broader public health, humanitarian, or economic crises, need to be disability-inclusive in order to prevent or reduce any disproportional impacts on PwD [1, 4, 5, 17, 26, 27].

In this context, this paper aims to present the PREparedness, RESponse, and SySTemic transformation (PRE-RE-SyST): a model for a disability-inclusive pandemic preparedness, responses, and systematic reduction of underlying disparities. The model was derived from a scoping review and thematic analysis of the literature on the disparities (i.e., disproportional impacts) experienced by PwD during the initial stages of the COVID-19 pandemic, most notably a review of the actions or recommended action to address these disparities.

## Methods

A scoping review and thematic analysis were used to build the PRE-RE-SyST model, i.e. the themes and sub-themes of the analysis provided the components for the PRE-RE-SyST model.

This paper articulates the final output of the scoping review of the literature on the disproportional impacts experienced by PwD during the initial stages of the COVID-19 pandemic, whose study protocol has been published [1]. From that review, we have published two initial papers outlining disproportional impacts experienced by PwD. One describes disproportional health risks and impacts of COVID-19 infections on PwD [18]. The other describes the broader health and social impact of lockdown measures on PwD [19]. In this final product, we provide an action model that thematically synthesizes recommended actions to address the previously identified disparities.

The Preferred Reporting Items for Systematic reviews and Meta-Analyses extension for Scoping Reviews (PRISMA-ScR) were used to guide the report of this scoping review [29].

### Eligibility criteria

Peer-reviewed empirical or perspective papers (including editorials or commentaries) or preprint empirical studies were included if they explicitly addressed: 1) the COVID-19 disease or pandemic, 2) PwD as a group, subgroup (e.g., based on impairment type or underlying diagnostic condition), or related individual circumstances as a pre-condition – i.e. impairments arising only as a consequence of the COVID-19 infection were excluded; 3) individual-level (e.g., health- or age-related) or social-level (e.g., healthcare access, living conditions) vulnerability to a COVID-19 infection or lockdown-related impact, and 4) action or recommended action reported to address any of the identified vulnerabilities or disparities.

Working definitions of PwD and vulnerability, including examples of individual and social-level vulnerabilities to the effects of the COVID-19 pandemic, were provided in the open-access study protocol, to support the reviewers in their eligibility decisions [1]. No geographic restrictions were applied. We considered papers in 6 languages (i.e., English, French, Spanish, Greek, Russian, and Portuguese), but after full-text assessments only English language papers met all our eligibility criteria.

### Information sources and search

A total of seven databases for the scientific, peer-reviewed literature (i.e., Medline/PubMed, Web of Science–Core Collection, Scopus, AgeLine, PsycINFO, CINAHL, and ERIC) were searched. As planned, [1] searches were run in mid-July 2020 and repeated 2 months later (i.e., in mid-September 2020), to cover data and perspectives from the first wave of the COVID-19 pandemic. As preprint databases hosted many studies that have not reached peer-reviewed publications during the initial stages of the COVID-19 pandemic, [30] we also searched three databases for preprint literature (i.e., MedRxiv, SocArXiv, and PsyArXiv), following the same process and dates. Before data charting, we searched for the peer-reviewed versions of any included preprints, and have replaced the record whenever found. The open-access protocol provides the full search details for each of the scientific and preprint databases [1].

A snowballing search process (e.g., citation tracking, referenced sources) was also conducted, using the included references to identify any additional records. Finally, supplied with a preliminary list of inclusions, members of the American Congress of Rehabilitation Medicine’s International Networking Group and Refugee Empowerment Task Force were consulted as key informants to provide any additional references.

Although planned, [1] we did not include elements of the grey literature (e.g., official reports from international organizations). During the initial searches, a living repository of that literature, hosted by the United Nations was found. That repository (https://www.un.org/development/desa/disabilities/covid-19.html) provides key grey literature resources from the United Nations, their specialty agencies, as well as from partner institutions (e.g., Disabled Person’s Organizations). To produce timely results as intended, [1] we opted to exclude the grey literature, collated elsewhere, and narrow the review coverage to the peer-reviewed literature and preprint studies. As scoping reviews map out initially unchartered territories, iterative decisions are common and acceptable, as long as they are reported and justified [31, 32].

### Selection process

Two independent reviewers (SK, SB) conducted the abstract-and-titles screenings and the full-text assessments against the eligibility criteria, after pilot screenings with over 80% agreements, overseen by the leading review author (TJ) [1]. Discrepancies were resolved through consensus or the leading author’s input.

### Data charting and items

Following a coding structure developed by members of the research team, one author (SK) extracted formal data elements (publication type, sources, geographies addressed), with a random 5% verified by another author (TJ). Regarding the content of the literature included, two independent reviewers (SK and SB) extracted text quotations on any action or recommended action to address disparities experienced by PwD related to the COVID-19 pandemic. These independent extractions from each paper were later paired for the qualitative data synthesis. The Additional file 1 provides these paired extractions and the reviewers’ brief synthesis of each paper.

### Critical appraisal

As planned [1] and as is common in scoping review methodologies, [31–33] no methodological quality assessments were performed.

### Synthesis

Descriptive statistics (e.g., percentages) were computed to provide a summative description of the amount and range of the related literature, including per publication type and source, country (or countries), or health conditions or impairments addressed. On the text quotations, we have developed a reflexive thematic analysis, [1, 34, 35] with a new interpretive schema for the emergent themes.

As planned, the thematic analysis was aimed to be theoretically-informed by an equity-oriented perspective, human-rights based perspectives, social and occupational justice lenses, universal design thinking, and systems-thinking approach [1]. Among them, a system-thinking approach [36–39] contributed to provide a structure for our analysis as these approaches can address the complex, perhaps even wicked, problem of disability disparities. The basic structure of the PRE-RE-SyST model was informed by systems thinking model in particular: the Iceberg Model [38, 40].

The Iceberg Model helps understand root, and often hidden, causes of an event (e.g., systemic structures and collective mental models such as cultural beliefs and assumptions) rather than merely the observable symptoms of that event (e.g., the pandemic-related disability disparities). The four levels in the Iceberg Model are: 1) ‘Events’: snapshot situations which are observable - for which one ‘Reacts’; 2) ‘Trends’: Patterns and behaviour over time - which one can ‘Anticipate’; 3) ‘Underlying Structures’: system components that influence the patterns – which one can ‘Design’ for; and 4) Mental models: Beliefs and assumptions that hold the system in place – which one can ‘Transform’ [38, 40]. The PRE-RE-SyST model was organized around a similar four-level understanding.

Within our PRE-RE-SyST model, we used ‘simples rules’ [41] for providing guidance, in the context of a complex and wicked problem. Initially used in the corporate world, ‘simple rules’ are principles-based, concrete and action-oriented guidelines (e.g., begin with action verbs), yet are not overly detailed, prescriptive, or cumbersome plans [41]. Hence, ‘simple rules’ aim to provide some flexibility in the way they are applied to complex adaptive systems (e.g., are adaptable to contexts, feedback loops, as well new opportunities as they arise), while maintaining consistency with the underlying principle. ‘Simple rules’ have been used in the development of landmark reports on tackling the healthcare quality chasm, nationally and globally [42, 43].

Finally, as planned, [1] we took a final consultation stage. Supplied with a preliminary version of the results and its discussion, members of the American Congress of Rehabilitation Medicine’s International Networking Group and Refugee Empowerment Task Force had the opportunity to comment and provide improvement suggestions over the preliminary results and their interpretation.

## Results

**Fig. 1:**
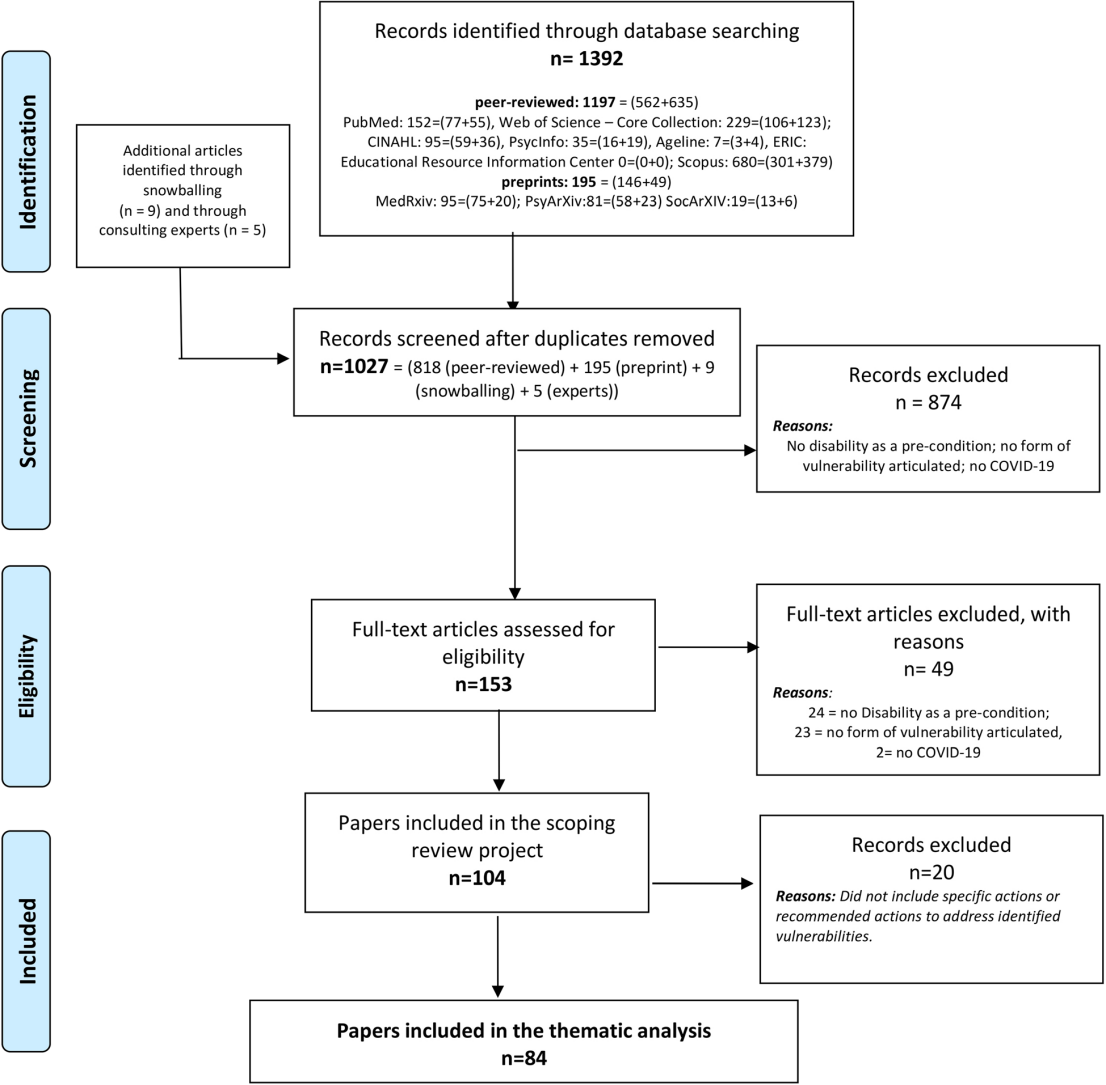
PRISMA flowchart of the scoping review with thematic analysis

The Fig. 1 provides a detailed flowchart of this review. In a synthesis, out of 1027 unique references, 153 underwent a full-text review, and 84 were included in the final analysis, i.e., report findings or rationales for any disproportionate, lockdown-related health or social consequences for PwD. The Additional file 1 lists the 84 papers included.

Among the 84 papers included, 22 (27%) were empirical studies (3 of which preprints), while the remaining were non-empirical (e.g., perspective papers, non-systematic reviews, experts recommendations). Fifty-two papers (62%) had no geographical focus (e.g., were applicable across locations). When they had a geographical focus, most (27 out of 32) addressed the United States (USA) or the United Kingdom (UK) experiences. While 33 papers (39%) addressed PwD overall (i.e., had no focus on specific health conditions or impairments), a sizeable amount addressed adults with cognitive impairments or intellectual disabilities (*n* = 17), children/youth with disabilities and their families (*n* = 10), and older adults experiencing disabilities (*n* = 9). The Additional file 1 provides the full distribution of the included paper per publication type, geographical focus, and health conditions addressed.

**Fig. 2:**
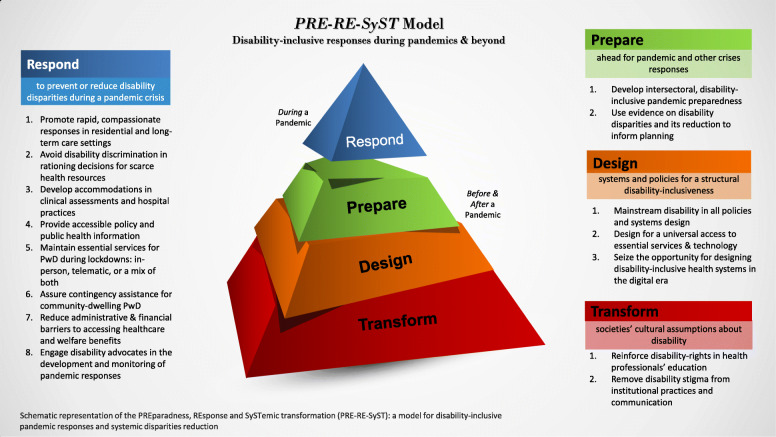
A schematic representation and overview of the PRE-RE-SyST model

Figure 2 provides a schematic representation and overview of the PRE-RE-SyST model. The central element of the figure is represented through a four-level pyramidal structure. Like in the Iceberg Model [38, 40], here the upper level represents the response to the current event, and the three lower levels represent responses to the major, underlying or root causes that are under the ‘surface’ and whose responses can provide deeper, transformative impacts. For each of the four levels, there is one major response. These reflect the four main themes (under colored background in the figure) of our thematic analyses. In turn, the sub-themes are provided in the form of ‘simple rules’ (numbered items in the figure). Within each ‘simple rule’, examples of actions are provided in the text below, which emerged from the literature reviewed.

Each main theme and then the ‘simple rules’ (i.e., sub-themes) within them are systematically described below. Of note, in addition to citing the 84 papers included in the scoping review, the initial scoping review results (i.e. two initial results papers from this project) are cited as well, i.e., they provide the context of the disparities addressed by the PRE-RE-SyST model

I) **Respond to prevent or reduce disability disparities during a pandemic crisis**

This main theme is focused on disability-inclusive responses during the pandemic event, and entails eight ‘simple rules’:

1. *Promote rapid, compassionate responses in residential and long-term care settings*

Rapid infection control practices might prevent or mitigate COVID-19 infections in residential and long-term care settings, where PwD are overrepresented and especially vulnerable to infection spreads and its health consequences [18]. For example, nursing and support practices on basic activities of daily living, which PwD often rely on, might be condensed and performed by the same practitioners, to reduce infection exposure and spread risks as much as possible [44].

Although rapidly enforced, infection control practices should be compassionate (i.e., provide an empathetic and humane approach), as well. For instance, pharmacological management, seclusion and other control measures to assure isolation (when less restrictive measures have failed) need to follow any form of guidelines that must be developed to avoid excessive or disproportionate uses of these measures [45]. Furthermore, isolation care plans for PwD might include what is known about the person (e.g. what the person enjoys doing), toward introducing favorite activities (e.g., colouring pages, playing movies and music on a tablet), coupled with signs for orientation [44, 45]. In interactions with people with cognitive or intellectual impairments, staff should introduce themselves when wearing personal protective equipment, may use a photograph in the protective clothing to help with identification, use the name of the person within the interaction, and continue to adopt a positive tone [46].

Similarly, stringent visitor policies may be adjusted for relatives of many PwD, under certain requirements, e.g., with negative virus testing, with transparent physical barriers, or through digital communication (e.g., videoconference). This aims to maintain social contact as well as facilitate communication of families with both staff and PwD, to prevent agitation and other psychological consequences in families and their loved ones [44, 47–51]. Finally, exemptions to stringent visitation policies might apply to families who wish to visit their loved ones in residential or long-term facilities at the end of life [52, 53].

2. *Avoid disability discrimination in rationing decisions for scarce health resources*

Impairments or disabilities are not akin to comorbidities, and do not affect medical outcomes per se [22, 23]. For example, people with Down’s syndrome may or may not have cardiac dysfunction, and only the latter can affect survival from the COVID-19; hence, while comorbidities may be a factor in any needed rationing of life-saving treatments, this applies across people, regardless the disability status [23]. Hence, in rationing decisions on life-saving treatments, it is useful to explicitly distinguish between disability status (e.g., deafness or intellectual impairments, which do not affect survival) and comorbidities that have been shown to affect survival (e.g., end-stage cancer, cardiac dysfunction) [23, 46, 54]. Overall, categorical exclusions, especially ones based on disability or diagnosis, have been discouraged [22]. In addition to basing decisions on objective medical assessments, information affecting survival of PwD and people overall (e.g. on whether to follow acute treatment or palliate care) should be individualized, preference-sensitive, and holistic [9, 22, 46].

Guidelines must explicitly state that disability should not be considered, otherwise any personal biases of medical staff or triage officers (e.g., on the appraised low quality of life or lower life worth of PwD) can put PwD at risk of prejudice and discrimination [23, 55, 56]. Scoring systems using quality-adjusted or disability-adjusted life-years should not be used too, as they are overtly discriminatory in explicitly counting a year for a person with disability as worth less than a year for an able-bodied person [22, 23]. For example, young people with stable long-term impairments but otherwise healthy can be unfairly disadvantaged [9]. Any form of discrimination against PwD is ethically reprehensible, against established human rights, as well as infringe upon antidiscrimination laws in place in many jurisdictions [11, 22, 24, 27, 54, 57–59].

Designating triage officers, training them to respect disability rights, [22, 24, 55] and/or ‘blinding’ them of all person’s characteristics not relevant to prognosis for hospital and near-term survival, can all help avoid disability prejudices [22]. Moreover, making rationing decisions collectively, e.g. by developing a triage committee to take or oversee decisions, can dilute or remove the effect of any personal bias on these important decisions; yet, the committee can still be ‘blinded’ to any medically irrelevant information, including disability status [11, 24, 54, 55, 58, 60]. Alternatively, these committees can include the presence or telematic consultation of ethicists, health professionals with a disability, disability scholars/advocates, rehabilitation professionals, or others, external or in-house, who are attuned with disability rights issues [11, 52, 54, 58]. Moreover, when PwD who use ventilators in their daily lives present to acute care hospital, their personal ventilators should not be reallocated to other patients [23, 61].

3. *Develop accommodations in clinical assessments and hospital practices*

Both clinical care and hospital practices may need accommodations to specific impairments or clinical manifestations of the COVID-19 on PwD [18].

For example, people with upper-level spinal cord injury might not be able to establish a productive cough, regardless of a respiratory infection [61, 62]. Similarly, clinical manifestations of the COVID-19 can be unusual among people with dementia (e.g., sometimes in the form of delirium rather than respiratory symptoms), requiring changes in medical assessment or monitoring practices [63]. A heightened suspicion index for COVID-19 infection should be developed, involving scientific experts to accommodate these and other specific symptoms, health risks of consequences for some PwD [61, 62]. The prompt involvement of healthcare professionals (e.g. in-house staff) with specific expertise in the disabling condition, whether in-person or virtual, can also be used in support of clinical care decisions for cases with new or worsening symptomatology of PwD, especially those with atypical manifestations [54, 61].

During hospitalizations due to COVID-19, virtual communication with families of PwD can help bridge many communication barriers between medical staff and a person with cognitive, intellectual, or communication impairments [44, 48, 55, 64]. In the interaction with persons with these impairments, speech of professionals could be slowed down as appropriate, but not condescending, and sentences should be as clear as possible, incorporating a single piece of information, i.e. avoiding complex and long sentences with multiple conjunctions and connective [46]. Like in long-term care settings, staff may hold a photograph in the protective clothing, introduce themselves, use the name of the person within the interaction, and adopt a positive and calming tone [46]. Plain-language forms might be used in brochures or other written material supplied at admission or discharge [58]. Finally, healthcare providers could also wear transparent masks to allow lip reading by people with hearing impairments [17].

4. *Provide accessible policy and public health information*

Press conferences and news broadcasts with public health and policy responses to the pandemic should be accessible to all, for example by including sign language interpreters [10, 17, 27, 65, 66]. Websites of official agencies, providing public health and policy resources, should fully comply with existing accessibility guidelines (e.g., of the World Wide Web Consortium); for example, the images of the text must be sufficiently differentiated so that users with some type of visual disability can differentiate the text when reading [67]. Pamphlets, brochures, outdoors and other forms of visual information might be designed to be accessible to people with low vision (e.g. with contrasts), and to be understandable by people with all literacy levels, including people with intellectual or learning disabilities [10, 17, 27, 46, 68–70]. Concrete public health recommendations are preferred than using concepts like ‘social distancing’. For example, instead of promoting ‘social distancing’, one can say: ‘you should go to the supermarket only once a week. Once you are there, avoid getting too close to the others’. [69] Non-governmental organizations have been producing support materials on COVID-19 and people with intellectual disabilities, targeting family carers, and voluntary groups and professionals, which have been disseminated in accessible formats [27, 71].

Accessible messages can be further circulated through multiple channels (e.g., email, text messaging, radio, television) [17, 58, 72]. Awareness programs on COVID-19 pandemic can also be created, to target vulnerable and at-risk populations, such as PwD and especially those from lower socioeconomic status, lower literacy level, and with poor social support [68–70]. Health and social services delivered to PwD should help disseminate knowledge and educate on specific protection measures, adapted to PwD and their caregivers [46, 71]. For example, people with visual impairments can be taught that their mobility long cane can be used to maintain the physical distance norm [70].

5. *Maintain essential services for PwD during lockdowns: in-person, telematic or a mix of both*.

PwD often rely on continuous health prevention, promotion, rehabilitation, or community-based services (e.g., outpatient, day services) to maintain or recover their health and function, and to prevent exacerbations of impairments or occurrence of secondary health conditions. Although substantively disrupted during initial lockdowns, [19] these services should be maintained as much as possible, and when not possible temporary substitutions should be explored.

For instance, these essential services might operate in person (with the due safety protections), through telematic means, or a tailored mix of both, which may include telematic consultations complemented by in-person care when hands-on approaches or heavy equipment are required [27, 47, 51, 60, 73–75]. Easily-accessible prescription refill for chronic medical conditions should be assured for PwD, at the same time that primary care services can have a role in screening for unmet healthcare needs [47]. Public health officials, peer-support networks, and other stakeholders might also encourage PwD and their caregivers to seek needed and available healthcare, even under lockdown conditions [63].

Telephone and online support systems, videoconferencing, technology-assisted platforms, or apps (promoting group exercise, relaxation, recreation activities, etc.) can be used to promote continued levels of daily (physical) activity, participation, life structure (e.g., balanced routine of activities), and needed social interaction [47, 53, 63, 76–81]. Promoting virtual gatherings, social chat rooms, online exercise and movie or game nights, etc. might also apply to prevent social isolation of community-dwelling PwD [48]. Phone-based solutions might be used for those without Internet access or digital literacy [82, 83].

Psychological and psychoeducational support or interventions for PwD and their informal caregivers should not be discontinued and often reinforced through lockdowns, even if delivered through telematic means [68, 76, 80, 84, 85]. For instance, children with disabilities and their parents need therapeutic, special education, psychosocial and other specialized services they often rely on, in-person or through telematic means; yet, with priority for being safely resumed in-person, as children with disabilities are disproportionally affected by school closures and disrupted routines [19, 76, 82, 86–88].

6. *Assure contingency assistance for community-dwelling PwD*

To prevent disruption of support networks, informal and formal caregivers of PwD, including personal assistants, can be considered “essential personnel”, [54, 65] thereby benefiting from additional work protections and special permission to commute, [70] and can be prioritized in the distribution of scarce health supplies (e.g. personal protective equipment, vaccines) [55].

Contingency responses are required for PwD whose formal or informal support networks have been disrupted due to lockdown or quarantine measures. Municipalities, social sector, civil society or other stakeholders might assure a community-level monitoring and support of PwD during lockdowns [27, 53, 65, 73]. For example, specially trained support workers can be made available 24/7 from a simple contact mechanism, either for planned or unplanned needs of PwD [74]. Support provided can include the delivery of basic goods (e.g., medications, food) at home [47, 53, 89]. Buzzers, alarms, helplines as well as emergency contact numbers might be available for PwD to use them in emergencies (e.g. falls, acute disease, maltreatment, or abuse) [47].

Community programs (e.g., parent or caregiver support groups) can proactively check in on families taking care of PwD (e.g., children with developmental disabilities) during lockdowns [86]. In turn, respite solutions should be provided as needed [76, 86, 87]. Additionally, outreach efforts might identify PwD that may be especially vulnerable (e.g., living alone, without caregiving support, at-risk children with disabilities), [88, 90] inclusively monitoring whether PwD are not being exploited, maltreated, or experiencing violence within the households during lockdowns [91]. For example, in Peru, measures were introduced to monitor the well-being of PwD in the community, thus ensuring not only access to health, but also protection from violence [4].

Finally, PwD living in the community can have protected hours to access essential services and shops (e.g., dentists, groceries) without risking navigating crowded spaces during pandemics [10, 48]. For example, a supermarket chain in Singapore dedicated hour-set aside for at-risk groups of people, including PwDs, 2 days per week, apart from the designated special checkout lines during normal operating hours [10].

7. *Reduce administrative & financial barriers to accessing healthcare and welfare benefits*

Telematic forms of service delivery are key for many PwD during lockdowns, but often hampered by licensing, reimbursement, or other administrative barriers, which could be eased during special (e.g. pandemic) times [92, 93]. For example, in the USA therapists managed to provide “e-visits” to already established patients, but struggled with the cost of the license for the telehealth platform [94]. In British Columbia, Canada, the healthcare system was implementing telemedicine billing codes to palliative care options during the pandemic for those living in long-term care facilities [52]. Furthermore, video conferencing consultations were implemented to enable that many acute-care services could be rather delivered in the long-term care setting [52]. Children with disabilities whose parents get suddenly unemployed and lost health insurance during the crises situations should continue to access healthcare they rely on [88]. Similarly, temporary waivers or extension of deadlines can be granted on the eligibility assessments for public-funded health insurance or health benefits [95].

Disability pensions and other welfare benefits might continue to be delivered (e.g. automatically maintained or renewed), in the context of greater difficulty to obtaining formal documentation [4]. These benefits may be even reinforced to offset the frequent reduced household income in a pandemic crises [4]. For example, many households of PwD in low- and middle-income countries (LMICs) only have one income, which can be threatened by pandemic-related unemployment [26]. This occurs in the context of increased costs of living for PWDs stemming from the pandemic, such as the extra costs of home deliveries and/or of hiring private support due to the suspension of public services [65]. One alternative would be to eliminate any restrictions governments might have on hiring family members to formally care for PwD [65]. Finally, in some LMICs, existing social protection programs which explicitly target PwD have been expanded during the pandemic [26].

8. *Engage disability advocates in the development and monitoring of pandemic responses*

Involving disability advocates in the design and monitoring of public health and policy responses to the COVID-19 pandemic is a key element to assure and promote the mainstreaming of disability rights in all response programs. These participatory approaches in policy design and implementation can avoid discriminatory practices, rights violation, and promote nimble adjustments in policies and practices to prevent health and social disparities [10, 27, 55, 56, 65, 96]. In one example, the early involvement of disability advocates in the development of triage and medical rationing guidelines could have prevented their unethical and illegal contents, which were only corrected a posteriori [22]. In another example, involvement of disability advocates in COVID-19 responses could have prevented unrestricted allowances for coffee shops to use up public space, such as pavements, turning urban landscapes even more inaccessible for PwD [4].

II) **Prepare ahead for pandemic and other crises responses**

Lack of disability-inclusive pandemic preparedness has been pointed as one of the main contributors to disability disparities in the pandemic responses [19].

1. *Develop intersectoral, disability-inclusive pandemic preparedness*

Proactive crisis preparedness is key to avoid discrimination of PwD during pandemic crises. Any of the pandemic responses mentioned above can more rapidly and effectively unfold in the presence of an intersectoral, disability-inclusive pandemic preparedness [55, 71, 72, 97].

Within jurisdictions, preparedness might imply defining the structures, process, stakeholders, and their responsibilities (e.g., for execution and monitoring pandemic responses). Once planned across societal sectors, disability-inclusive pandemic responses can be rapidly activated to address pandemic situations [48, 55, 56]. Within sectors or institutions, long-term care settings, in which PwD are overrepresented and infection risks are greater, should develop comprehensive outbreak preparedness, including employees’ training [49, 55].

Finally, disability advocates must be involved in the mainstream pandemic or emergency preparedness, to assure that preparedness is truly disability-inclusive. A full involvement and representation from the disability community is crucial for a disability-inclusive pandemic preparedness [11, 26, 72].

2. *Use evidence on disability disparities and its reduction to inform planning*

Disability-inclusive preparedness plans can be more effective or precise if they rely on evidence, either quantitative or qualitative, on the disability-related disparities and on ways to promote their reduction. Numerous calls have been made for the development of research that can further highlight the challenges and experiences of PwD in the current COVID-19 pandemic, and to learn about individual-, family-, community- and population-level interventions that can be included in the preparedness for pandemic responses [20, 47, 48, 55, 98–100].

In addition to collecting, synthesizing, and using existing disability-related evidence for pandemic responses and preparedness, that body of knowledge needs to be purposely built [27, 101]. For instance, public health agencies and healthcare institutions might systematically collect and report dis-aggregated data among PwD (e.g. on infection trends) [20, 48, 99]. Sometimes, this may take small but significant changes in data collection, such a systematically recording any disability status on deaths certificates [100]. Challenges experienced during lockdowns might be determined for specific sectors or subpopulations, such as children with disabilities, because it is important to understand how essential services (e.g. school, therapies) can be safely maintained or rapidly resumed, to be applicable to future lockdowns [88, 102]. Overall, disability-disaggregated data and research would better inform policy and program development and preparedness to prevent aggravation of any existing disparities experienced by PwD during pandemic events [11].

III) **Design systems and policies for a structural disability-inclusiveness**

Pandemic disparities experienced by PwD were found to reflect exacerbations of socially-determined disparities PwD have been experiencing for long time [19]. Hence, the opportunity lies on a fundamental (re-)design of systems, sectors, and policies for a structural disability-inclusiveness. This would address the root causes of pandemic disparities as well as systematically reduce inequalities that PwD have been experiencing, everyday [72]. If disability-inclusiveness becomes as system’s property (i.e., design feature), it can also become the permanent standard – for pandemics and beyond.

1. *Mainstream disability in all policies and systems design*

PwD is a minority group often marginalized and segregated from mainstream policy-making, yet universal design principles can apply to health, social, or development policies [26]. For instance, PwD also should be included in the medium and long-term recovery plans once the immediate global public health and financial crisis subsides [4]. Formal inclusion of disability perspectives in institutional and governmental decision-making bodies should be instrumental to this concept [55].

Disability-inclusive policies, in turn, should be imprinted into antidiscrimination laws and regulations, whose enforcement needs to be strengthened as part of a broader human-rights approach [65, 73]. During the pandemic, some PwD have noticed that legally-entitled accommodations they had been asking for long time (e.g. flexible work and study from home options) were addressed almost overnight, and without the need for invasive documentation, accountability, and control systems [10]. This occurred as telework accommodations have become commonplace across larger segments of the population, which emphasized that there is room for these (legally-required) accommodations for PwD to become everyday practices [10, 72]. Welfare and other policies should also be deployed in a way that makes them accessible to all those eligible, including PwD. In one example, the streamlining and decentralization of the application processes for social benefits in LMICs was credited with increasing enrolment in disability-targeted programs [26].

To complement mainstreaming policies, disability-specific policies should also address specific needs of PwD. Overall, a twin track approach is recommended: PwD need to be considered both in mainstream policy and in disability-specific policy, [4] the latter compensating for special needs of PwD (e.g. any transportation and assistance support, extra costs of living with a disability), which provide social disadvantages if not addressed [4]..

2. *Design for a universal access to essential services & technology*

Universal design principles might be enforced to equitably promote everyone’s access to essential services and products across sectors. This notion goes well beyond architecture and urban planning, and progressively involves digital and technological solutions, as society increasingly relies on them. Only accelerated by the pandemic, digital and technological solutions have been increasingly used as an alternative or complementary way to obtain everyday health, educational, and other public or private services. These are now available through websites, apps, videoconferencing, telehealth, e-learning, net-banking, or e-commerce platforms. PwD cannot be fully integrated in society if these and other digital solutions and technologies are not disability-inclusive by design, e.g., if they don’t provide accessibility options embedded in a system [88, 96].

For that to occur, PwD need to be routinely engaged in the development and testing of any new solutions or technologies. For example, PwD (e.g. with cognitive impairments from dementia) might be engaged in the user-testing of remotely-delivered care or support interventions [103]. Similarly, digital solutions (e.g., apps) that promote sports and physical activity, including during lockdown periods, should be inclusive of PwD [104].

Accessibility options in software should be mainstreamed and not come as an additional cost for PwD [105]. When not accessible by some PwD, either due to any impairments or social circumstances, essential services delivered remotely might include options for the use of low-cost and simpler technology such as landline, cellular phones, and text messaging, with simple messages - to avoid leaving people behind [47, 106, 107]. Finally, disability-specific accommodations in hardware (e.g. bio-peripheral devices that compensate for physical mobility problems), or overall assistive devices products, should be universally accessible to promote and facilitate a digital inclusion and societal inclusion of PwD, rather than deepening or accentuating a digital divide [105].

Programs that overcome digital literacy gaps for PwD or provide assistance on their use can also help overcome the digital divide [47, 79, 107]. PwD can be trained on how to independently and safely access abuse-related resources, such as using an incognito browser and immediately clearing browser history, overcoming the perpetrator’s supervision [108].

Finally, in addition to accessible mainstream platforms, digital and technological solutions (e.g. online support resources, augmented reality innovations, robots, television-based assistive integrated service) should specifically target unique needs of PwD (e.g. with visual or mild cognitive impairments living in the community), promoting independence, physical activity, and social interaction in both pandemic and non-pandemic times [50, 81, 109–111].

3. *Seize the opportunity for designing disability-inclusive health systems in the digital era*

While technology and digital inclusion of PwD is needed across sectors, heath-sector-specific opportunities must be seized for systematically preventing or reducing disability health and healthcare disparities.

For instance, in addition to functional information on disability identifiers (e.g. omitted for consultation if not relevant for the task), electronic health records can incorporate relevant environmental-level information, including social determinants of health [112]. This can provide a broader picture of the client and populations served and readily available data for identifying health and healthcare disparities experienced by PwD, in both pandemic and non-pandemic times [48, 98, 99]. Up-to-date hospital ‘passports’ for PwD (e.g. with cognitive, intellectual, developmental, or communication impairments) can also be included in electronic health records, to provide information about the person, clinical and about personal preferences, that may inform more effective and compassionate care [46].

In turn, digital or tele-health platforms should include disability-friendly accessibility options, for example: remote audiovisual description services for blind and low-vision individuals, and captioning or third-party remote connection with American Sign Language interpreter for deaf and hard-of-hearing people [54]. By design, these platforms should be simple and intuitive to use by person with low digital and health literacy [81, 93]. Not the least, all individuals interacting with PwD through a telehealth portal must be trained on how do so [86, 113].

Telehealth regulatory frameworks need to be permanently legislated and coherent, instead of conflicting local, state, insurance, and federal regulations [105]. Lack of coherent regulatory plans can significantly impede relevant care and support for PwD provided across jurisdictions [113]. Additionally, reimbursement plans might cover needed telehealth or telerehabilitation solutions that PwD may need to rely on [114]. Many PwD could benefit from these solutions in the post-pandemic period, as a useful adjunct for any in-person care, [75, 86, 105] for example, in combination with outpatient or home-based interventions [92].

The increased, complementary use of technologies and digital health services by PwD can bring several advantages. In addition to more frequent interactions or monitoring activities (e.g. of physical activity through the use of accelerometers [84]), benefits might include reduction in non-essential travel at the backdrop of sometimes complex transportation needs [79, 115]. Also, PwD could participate in wider, more geographically dispersed support and peer-support communities [79]. Overall, many of the challenges to the start-up and maintenance of digital health solutions may have been overcome during pandemic times, out of necessity, and should be further developed for more inclusive health systems [72, 79, 116].

IV) **Transform society’s cultural assumptions about disability**

Pandemic or emergency disparities, as well as everyday disparities experienced by PwD, have root causes in biased cultural beliefs and assumptions which lead to disability stigma and thinking in ableist ways, which are then reflected in discrimination, social disadvantage, prejudice, or marginalization of PwD [19]. Addressing these root causes and transforming society’s cultural assumptions about disability is, therefore, part of a systematic, continuous approach to reduce socially-entrenched disparities experienced by PwD experience, in both pandemic and non-pandemic times.

1. *Reinforce disability-rights in health professional’s education*

Systemic discrimination stemming from pervasive attitudes and ableism of healthcare professionals should be eliminated [11, 24, 100]. educating health professionals on disability rights is required to reduce the effect of personal bias on key healthcare decisions, which can apply in everyday practices and in crisis periods, when rationing decisions may take place. Indeed, while this training for healthcare professional may be impractical during busy pandemic times, it should be mandatory for long-term disability disparities reduction [54, 58].

Importantly, healthcare providers should receive education about disability not simply related to bio-medical topics but also as a social and political experience, and learn to become aware of and question their biases and assumptions about quality of life among PwD [11]. Humanities training at all levels of education, involving ethical reflection and critical thinking skills may be one solution [112]. Healthcare professionals, especially those who commonly interact with PwD, should be educated for any prevalent, socially-driven disability disparities (e.g. abuse and maltreatment) and trained to advocate for PwD to address these abuses [108].

2. *Remove disability stigma from institutional practices and communication*

For a lasting transformation, all societal manifestations of ableism and disability stigma should be avoided. This process can start with stakeholders accountable for formal institutional communication, either public or private, which can be translated to institutional bills of rights, professional ethics codes, and broadly all forms of communication disseminated to employees, constituencies, served populations, and the society in general [55, 59]. Widely established discriminatory practices should also be challenged, and replaced with the implementation of new, more inclusive cultural models. For example, the enrolment procedures in health research must be adapted to be more inclusive of PwD [26].

Finally, formal inclusion of disability advocates in institutional and governmental decision-making bodies can be important to influence a more inclusive societal thinking and practices, starting at the policy-making level [55].

## Discussion

This paper presents the PRE-RE-SyST, a model for disability-inclusive pandemic responses and systemic disparities reduction for PwD. The model was derived from a scoping review and thematic analysis of the literature on recommended actions to address health and social disparities experienced by PwD, during the initial stages of the COVID-19 pandemic, i.e., literature reviewed up to mid-September 2021.

The theoretical basis of the PRE-RE-SyST model is a systems-thinking approach grounded in equity and human rights, universal design principles, and social and occupational justice perspectives, as explained in the study protocol [1]. Specifically, the Iceberg Model [38, 40] provided the structure for collating and organizing the themes that emerged from the literature. Indeed, the emergent themes addressed disability disparities observable during the pandemic event, but also three levels of root, structural, and systemic causes that lay under the surface: lack of disability-inclusive pandemic preparedness, lack of everyday disability-inclusive systems, policies and practices, and discriminatory cultural assumptions about disability prevalent in societies.

Moreover, the PRE-RE-SyST model follows the assumption that disability disparities, exacerbated and made more visible during the COVID-19 pandemic, can foster greater social awareness and provide opportunities for change towards a more inclusive society, after the pandemic [72, 117]. This assumption is aligned with the “build back better” framework which positions disasters as an opportunity to build more resilient systems, [118] as well as the more recent “build back fairer” framework, aimed at breaking cycles of inequality [119, 120]. The latter fosters the adoption of equity-oriented approaches to population health and development toward building more resilient societies, better prepared to weather future pandemics and other emergencies (e.g. natural disasters, humanitarian and financial crises) [119, 120]. More than bouncing back from a crisis, societal systems, such as healthcare, need to address ongoing and structural strains toward building ‘everyday resilience’ [121]. The PRE-RE-SyST model assumes that disability-inclusiveness is everyone’s business, requires mainstream action, and should be fostered everyday, i.e., not only during pandemics.

Within each of the four main themes, the PRE-RE-SyST model provides actionable guidance via ‘simple rules’ [41]. These rules provide concrete, yet adaptive, guidance to inform action by multiple stakeholders (e.g., policy-makers, public health authorities, civil society) across diverse contexts and evolving problems. Hence, although actionable (e.g., starting with action verbs), the ‘simple rules’ provided here were not overly detailed, and deliberately so. ‘Simple rules’ can accommodate innovative approaches as well as should be adaptive to emergent problems. For example, within the ‘simple rule’ to “*avoid disability discrimination in rationing decisions for scarce health resources*”, we provide examples of action related to scarce acute-care resources such as ventilators (i.e., the prevalent issue in the literature up to mid-September 2020). The ‘simple rule’, though, can be applied to inform decision-making of other scarce health resources, such as COVID-19 vaccines.

Indeed, the prioritization of the access to COVID-19 vaccines should follow a human-rights approach, considering: (1) infection risk and severity of pre-existing diseases; (2) social vulnerabilities; and (3) potential financial and social effects of ill health [122]. With that framework, many PwD (e.g. living in congregated settings, with comorbid conditions, unable to comply with preventive measures) should be among those prioritized. The WHO’s guidelines for a COVID-19 vaccine allocation strategy includes PwD, as a socio-demographic group, among those to be vaccinated when the supply is limited (e.g., only sufficient to cover 11–20% of the population) [123]. Similarly, multidisciplinary committees as well as lawyers have been arguing that PwD should have priority access to vaccines [124, 125]. Finally, formal and informal caregivers of PwD might also be considered as a priority - in order to avoid disruption of key services and support many PwD rely on for fulfilling basic needs [19].

Among the disability disparities that have been exacerbated during the COVID-19 pandemic, [18, 19, 126] the digital divide [19] is one especially addressed by the PRE-RE-SyST model, notably at the ‘design’ level. During the COVID-19 pandemic, this issue has prevented many PwD to access essential services, because digital solutions were not disability-inclusive. The lack of digital solutions that are disability-inclusive by design is not a new problem, rather it is a systemic one and affects PwD’s societal participation in much broader terms, especially as technology and digital solutions play a larger role in societies [127–129].

Finally, following the widely known premise in disability advocacy of “nothing for us without us”, [55] the PRE-RE-SyST model encourages, across its levels, the involvement of PwD and/or their advocates as one means to ensure that PwD are not ‘left behind’ [27, 66].

### Limitations

This paper should be interpreted in light of the following limitations.

This systematic review addressed the preprint and peer-reviewed literature, including non-empirical papers, available only up to mid-September 2020, thereby reporting recommended actions from the first wave of the COVID-19 pandemic. Also, the synthesized recommended actions do not equate to action that has been proven effective to address disability disparities. In fact, one ‘simple rule’ was built around the need to further develop and systematically “*use evidence on disability disparities and its reduction to inform planning*”.

The PRE-RE-SyST model does not distinguish among higher or lower income countries or communities. Therefore the operationalization of the ‘simple rules’ may differ across contexts as local structures, cultures, and resources vary. While specific solutions may differ, and PwD in lower income nations may face a greater magnitude of disparities and social vulnerabilities, disability disparities, during and previous to the COVID-19 pandemic, have been found within every context [6, 7, 18, 19, 130]. Hence, the PRE-RE-SyST model, notably its four-level themes and the ‘simple rules’ within, has the potential to be implemented everywhere, provided that it be applied in a context-sensitive manner by local policy-makers, public health officials, or any supervisory committees, inclusive of disability advocates.

## Conclusion

The PRE-RE-SyST model articulates main strategies and ‘simple rules’ (i.e., the elements displayed in Fig. 1), as well as possible means (i.e., examples of recommended action extracted from the literature, described throughout our results) whereby public health authorities, policy-makers, and other stakeholders can systematically address or prevent disability disparities in pandemic crises and similar emergencies. Specifically, we present eight ‘simple rules’ for action on responding to prevent or reduce disability disparities during a pandemic crisis (e.g., avoid disability discrimination in rationing decisions for scarce health resources), two ‘simple rules’ for stakeholders to prepared ahead for pandemic and other crises responses (e.g. use evidence on disability disparities and its reduction to inform planning), three ‘simple rules’ on the design of systems and policies for a structural disability-inclusiveness (e.g. mainstream disability in all policies and systems design), and three ‘simple rules’ on transforming societies’ cultural assumptions about disability (e.g. reinforce disability-rights in health professionals’ education). As a whole, the PRE-RE-SyST model can be used to foster equity-oriented policies and practices to transform and improve societies’ overall resilience to pandemics and other public health emergencies, in a disability-inclusive manner. In addition to pandemic responses, disability-inclusiveness is needed everyday, from policy and systems design to transformational changes in cultural beliefs and assumptions - applicable to pandemics and beyond.

## Supplementary Information

**Additional file 1.**

## Data Availability

All the data and materials of this study is either incorporated within this publication or made available for open access in previously published literature with the related links provided in this publication.
