# Supplementary material for: PREparedness, REsponse and SySTemic transformation (PRE-RE-SyST): a model for disability-inclusive pandemic responses and systemic disparities reduction derived from a scoping review and thematic analysis
Source: Int J Equity Health. 2021 Sep 14;20:204. doi: 10.1186/s12939-021-01526-y (PMC8438560; doi:10.1186/s12939-021-01526-y)
Supplement: Supplementary file 1 — Additional file 1. [file 12939_2021_1526_MOESM1_ESM.zip › Web-appendix 2 - list of papers included (3).docx]

**Web-Appendix**

List of papers included

Aishworiya, R., & Kang, Y. Q. (2020). Including Children with Developmental Disabilities in the Equation During this COVID-19 Pandemic. *J Autism Dev Disord*, 1-4. <https://doi.org/10.1007/s10803-020-04670-6>

Alexander, R., Ravi, A., Barclay, H., Sawhney, I., Chester, V., Malcolm, V., Brolly, K., Mukherji, K., Zia, A., Tharian, R., Howell, A., Lane, T., Cooper, V., & Langdon, P. E. (2020). Guidance for the Treatment and Management of COVID-19 Among People with Intellectual Disabilities. *J Policy Pract Intellect Disabil*. <https://doi.org/10.1111/jppi.12352>

Andrews, E. E., Ayers, K. B., Brown, K. S., Dunn, D. S., & Pilarski, C. R. (2020). No body is expendable: Medical rationing and disability justice during the COVID-19 pandemic. *Am Psychol*. <https://doi.org/10.1037/amp0000709>

Annaswamy, T. M., Verduzco-Gutierrez, M., & Frieden, L. (2020). Telemedicine barriers and challenges for persons with disabilities: Covid-19 and beyond. *Disabil Health J*, 100973. <https://doi.org/10.1016/j.dhjo.2020.100973>

Armitage, R., & Nellums, L. B. (2020). The COVID-19 response must be disability inclusive. *Lancet Public Health*, *5*(5), e257. <https://doi.org/10.1016/s2468-2667(20)30076-1>

Asbury, K., Fox, L., Deniz, E., Code, A., & Toseeb, U. (2020). How is COVID-19 Affecting the Mental Health of Children with Special Educational Needs and Disabilities and Their Families? *J Autism Dev Disord*, 1-9. <https://doi.org/10.1007/s10803-020-04577-2>

Azarpazhooh, M. R., Amiri, A., Morovatdar, N., Steinwender, S., Rezaei Ardani, A., Yassi, N., Biller, J., Stranges, S., Tokazebani Belasi, M., Neya, S. K., Khorram, B., Sheikh Andalibi, M. S., Arsang-Jang, S., Mokhber, N., & Di Napoli, M. (2020). Correlations between COVID-19 and burden of dementia: An ecological study and review of literature. *J Neurol Sci*, *416*, 117013. <https://doi.org/10.1016/j.jns.2020.117013>

Balestrini, S., Koepp, M. J., Gandhi, S., Rickman, H., Shin, G. Y., Houlihan, C., Anders-Cannon, J., Silvennoinen, K., Xiao, F., Zagaglia, S., Hudgell, K., Ziomek, M., Haimes, P., Sampson, A., Parker, A., Cross, J. H., Pardington, R., Nastouli, E., Swanton, C., Sander, J. W., & Sisodiya, S. (2020). Clinical outcomes of SARS-CoV-2 pandemic in long-term care facilities for people with epilepsy: observational study. *medRxiv*, 2020.2006.2010.20123281. <https://doi.org/10.1101/2020.06.10.20123281>

Banks, L. M., Davey, C., Shakespeare, T., & Kuper, H. (2021). Disability-inclusive responses to COVID-19: Lessons learnt from research on social protection in low- and middle-income countries. *World Dev*, *137*, 105178. <https://doi.org/10.1016/j.worlddev.2020.105178>

Banskota, S., Healy, M., & Goldberg, E. M. (2020). 15 Smartphone Apps for Older Adults to Use While in Isolation During the COVID-19 Pandemic. *West J Emerg Med*, *21*(3), 514-525. <https://doi.org/10.5811/westjem.2020.4.47372>

Ben-Pazi, H., Beni-Adani, L., & Lamdan, R. (2020). Accelerating Telemedicine for Cerebral Palsy During the COVID-19 Pandemic and Beyond. *Front Neurol*, *11*, 746. <https://doi.org/10.3389/fneur.2020.00746>

Benaque, A., Gurruchaga, M. J., Abdelnour, C., Hernández, I., Cañabate, P., Alegret, M., Rodríguez, I., Rosende-Roca, M., Tartari, J. P., Esteban, E., López, R., Gil, S., Vargas, L., Mauleón, A., Espinosa, A., Ortega, G., Sanabria, A., Pérez, A., Alarcón, E., González-Pérez, A., Marquié, M., Valero, S., Tárraga, L., Ruiz, A., & Boada, M. (2020). Dementia Care in Times of COVID-19: Experience at Fundació ACE in Barcelona, Spain. *J Alzheimers Dis*, *76*(1), 33-40. <https://doi.org/10.3233/jad-200547>

Bhaskar, S., Rastogi, A., Chattu, V. K., Adisesh, A., Thomas, P., Alvarado, N., Riahi, A. D., Varun, C. N., Pai, A. R., Barsam, S., & Walker, A. H. (2020). Key Strategies for Clinical Management and Improvement of Healthcare Services for Cardiovascular Disease and Diabetes Patients in the Coronavirus (COVID-19) Settings: Recommendations From the REPROGRAM Consortium. *Front Cardiovasc Med*, *7*, 112. <https://doi.org/10.3389/fcvm.2020.00112>

Blewett, L. A., & Hest, R. (2020). Emergency Flexibility for States to Increase and Maintain Medicaid Eligibility for LTSS under COVID-19. *J Aging Soc Policy*, *32*(4-5), 343-349. <https://doi.org/10.1080/08959420.2020.1774312>

Boyle, C. A., Fox, M. H., Havercamp, S. M., & Zubler, J. (2020). The public health response to the COVID-19 pandemic for people with disabilities. *Disabil Health J*, *13*(3), 100943. <https://doi.org/10.1016/j.dhjo.2020.100943>

Brown, E. E., Kumar, S., Rajji, T. K., Pollock, B. G., & Mulsant, B. H. (2020). Anticipating and Mitigating the Impact of the COVID-19 Pandemic on Alzheimer's Disease and Related Dementias [Article]. *American Journal of Geriatric Psychiatry*, *28*(7), 712-721. <https://doi.org/10.1016/j.jagp.2020.04.010>

Capozzo, R., Zoccolella, S., Musio, M., Barone, R., Accogli, M., & Logroscino, G. (2020). Telemedicine is a useful tool to deliver care to patients with Amyotrophic Lateral Sclerosis during COVID-19 pandemic: results from Southern Italy. *Amyotroph Lateral Scler Frontotemporal Degener*, 1-7. <https://doi.org/10.1080/21678421.2020.1773502>

Chase, J. (2020). Caring for Frail Older Adults During COVID-19: Integrating Public Health Ethics into Clinical Practice. *J Am Geriatr Soc*, *68*(8), 1666-1670. <https://doi.org/10.1111/jgs.16666>

Chen, B., & McNamara, D. M. (2020). Disability Discrimination, Medical Rationing and COVID-19. *Asian Bioeth Rev*, 1-8. <https://doi.org/10.1007/s41649-020-00147-x>

Cheung, G., & Peri, K. (2020). Challenges to dementia care during COVID-19: Innovations in remote delivery of group Cognitive Stimulation Therapy. *Aging Ment Health*, 1-3. <https://doi.org/10.1080/13607863.2020.1789945>

Courtenay, K., & Perera, B. (2020). COVID-19 and people with intellectual disability: impacts of a pandemic. *Ir J Psychol Med*, *37*(3), 231-236. <https://doi.org/10.1017/ipm.2020.45>

Cox, D. J., Plavnick, J. B., & Brodhead, M. T. (2020). A Proposed Process for Risk Mitigation During the COVID-19 Pandemic. *Behav Anal Pract*, *13*(2), 1-7. <https://doi.org/10.1007/s40617-020-00430-1>

D'Cruz, M., & Banerjee, D. (2020). 'An invisible human rights crisis': The marginalization of older adults during the COVID-19 pandemic - An advocacy review. *Psychiatry Res*, *292*, 113369. <https://doi.org/10.1016/j.psychres.2020.113369>

Devita, M., Bordignon, A., Sergi, G., & Coin, A. The psychological and cognitive impact of Covid-19 on individuals with neurocognitive impairments: research topics and remote intervention proposals. *Aging Clinical and Experimental Research*. <https://doi.org/10.1007/s40520-020-01637-6>

Eghtesadi, M. (2020). Breaking Social Isolation Amidst COVID-19: A Viewpoint on Improving Access to Technology in Long-Term Care Facilities. *J Am Geriatr Soc*, *68*(5), 949-950. <https://doi.org/10.1111/jgs.16478>

Eskytė, I., Lawson, A., Orchard, M., & Andrews, E. (2020). Out on the Streets - Crisis, Opportunity and Disabled People in the Era of Covid-19: Reflections from the UK. *Alter*. <https://doi.org/10.1016/j.alter.2020.07.004>

Fallon, N., Brown, C., Twiddy, H., Brian, E., Frank, B., Nurmikko, T., & Stancak, A. (2020). Adverse effects of COVID-19 related lockdown on pain, physical activity and psychological wellbeing in people with chronic pain. *medRxiv*, 2020.2006.2004.20122564. <https://doi.org/10.1101/2020.06.04.20122564>

Farrell, T. W., Ferrante, L. E., Brown, T., Francis, L., Widera, E., Rhodes, R., Rosen, T., Hwang, U., Witt, L. J., Thothala, N., Liu, S. W., Vitale, C. A., Braun, U. K., Stephens, C., & Saliba, D. (2020). AGS Position Statement: Resource Allocation Strategies and Age-Related Considerations in the COVID-19 Era and Beyond. *J Am Geriatr Soc*, *68*(6), 1136-1142. <https://doi.org/10.1111/jgs.16537>

Fazzi, E., & Galli, J. (2020). New clinical needs and strategies for care in children with neurodisability during COVID-19. *Dev Med Child Neurol*, *62*(7), 879-880. <https://doi.org/10.1111/dmcn.14557>

Fegert, J. M., Vitiello, B., Plener, P. L., & Clemens, V. (2020). Challenges and burden of the Coronavirus 2019 (COVID-19) pandemic for child and adolescent mental health: a narrative review to highlight clinical and research needs in the acute phase and the long return to normality. *Child Adolesc Psychiatry Ment Health*, *14*, 20. <https://doi.org/10.1186/s13034-020-00329-3>

Fernández-Díaz, E., Iglesias-Sánchez, P. P., & Jambrino-Maldonado, C. (2020). Exploring WHO Communication during the COVID 19 Pandemic through the WHO Website Based on W3C Guidelines: Accessible for All? *Int J Environ Res Public Health*, *17*(16). <https://doi.org/10.3390/ijerph17165663>

Fitzgerald, H., Stride, A., & Drury, S. (2020). COVID-19, lockdown and (disability) sport. *Managing Sport and Leisure*, 1-8. <https://doi.org/10.1080/23750472.2020.1776950>

Frederick, J. K., Raabe, G. R., Rogers, V. R., & Pizzica, J. (2020). Advocacy, collaboration, and intervention: A model of distance special education support services amid covid-19. *Behav Anal Pract*. <https://doi.org/10.1007/s40617-020-00476-1>

Frost, R., Nimmons, D., & Davies, N. (2020). Using Remote Interventions in Promoting the Health of Frail Older Persons Following the COVID-19 Lockdown: Challenges and Solutions. *J Am Med Dir Assoc*, *21*(7), 992-993. <https://doi.org/10.1016/j.jamda.2020.05.038>

Goggin, G., & Ellis, K. (2020). Disability, communication, and life itself in the COVID-19 pandemic. *Health Sociology Review*, *29*(2), 168-176. <https://doi.org/10.1080/14461242.2020.1784020>

Goodman-Casanova, J. M., Dura-Perez, E., Guzman-Parra, J., Cuesta-Vargas, A., & Mayoral-Cleries, F. (2020). Telehealth Home Support During COVID-19 Confinement for Community-Dwelling Older Adults With Mild Cognitive Impairment or Mild Dementia: Survey Study. *J Med Internet Res*, *22*(5), e19434. <https://doi.org/10.2196/19434>

Guidry-Grimes, L., Savin, K., Stramondo, J. A., Reynolds, J. M., Tsaplina, M., Burke, T. B., Ballantyne, A., Kittay, E. F., Stahl, D., Scully, J. L., Garland-Thomson, R., Tarzian, A., Dorfman, D., & Fins, J. J. (2020). Disability Rights as a Necessary Framework for Crisis Standards of Care and the Future of Health Care. *Hastings Cent Rep*, *50*(3), 28-32. <https://doi.org/10.1002/hast.1128>

Hoffman, G. J., Webster, N. J., & Bynum, J. P. W. (2020). A Framework for Aging-Friendly Services and Supports in the Age of COVID-19. *J Aging Soc Policy*, *32*(4-5), 450-459. <https://doi.org/10.1080/08959420.2020.1771239>

Iaboni, A., Cockburn, A., Marcil, M., Rodrigues, K., Marshall, C., Garcia, M. A., Quirt, H., Reynolds, K. B., Keren, R., & Flint, A. J. (2020). Achieving Safe, Effective, and Compassionate Quarantine or Isolation of Older Adults With Dementia in Nursing Homes. *Am J Geriatr Psychiatry*, *28*(8), 835-838. <https://doi.org/10.1016/j.jagp.2020.04.025>

Iob, E., Steptoe, A., & Fancourt, D. (2020). Abuse, self-harm and suicidal ideation in the UK during the COVID-19 pandemic. *Br J Psychiatry*, *217*(4), 543-546. <https://doi.org/10.1192/bjp.2020.130>

Jesus, T. S., Landry, M. D., & Jacobs, K. (2020). A 'new normal' following COVID-19 and the economic crisis: Using systems thinking to identify challenges and opportunities in disability, telework, and rehabilitation. *Work*, *67*(1), 37-46. <https://doi.org/10.3233/wor-203250>

Korupolu, R., Stampas, A., Gibbons, C., Hernandez Jimenez, I., Skelton, F., & Verduzco-Gutierrez, M. (2020). COVID-19: Screening and triage challenges in people with disability due to Spinal Cord Injury. *Spinal Cord Ser Cases*, *6*(1), 35. <https://doi.org/10.1038/s41394-020-0284-7>

Kuper, H., Banks, L. M., Bright, T., Davey, C., & Shakespeare, T. (2020). Disability-inclusive COVID-19 response: What it is, why it is important and what we can learn from the United Kingdom's response. *Wellcome Open Res*, *5*, 79. <https://doi.org/10.12688/wellcomeopenres.15833.1>

Landes, S. D., Turk, M. A., Formica, M. K., McDonald, K. E., & Stevens, J. D. (2020). COVID-19 outcomes among people with intellectual and developmental disability living in residential group homes in New York State. *Disabil Health J*, *13*(4), 100969. <https://doi.org/10.1016/j.dhjo.2020.100969>

Landi, D., Ponzano, M., Nicoletti, C. G., Cecchi, G., Cola, G., Mataluni, G., Mercuri, N. B., Sormani, M. P., & Marfia, G. A. (2020). Adherence to social distancing and use of personal protective equipment and the risk of SARS-CoV-2 infection in a cohort of patients with multiple sclerosis. *Mult Scler Relat Disord*, *45*, 102359. <https://doi.org/10.1016/j.msard.2020.102359>

Lee, O. E., & Davis, B. (2020). Adapting 'Sunshine,' A Socially Assistive Chat Robot for Older Adults with Cognitive Impairment: A Pilot Study. *J Gerontol Soc Work*, 1-3. <https://doi.org/10.1080/01634372.2020.1789256>

Lee, S., & Kim, J. (2020). A country report: Impact of covid-19 and inequity of health on south korea’s disabled community during a pandemic. *Disability & Society*. <https://doi.org/10.1080/09687599.2020.1809352>

Lim, W. S., Liang, C. K., Assantachai, P., Auyeung, T. W., Kang, L., Lee, W. J., Lim, J. Y., Sugimoto, K., Akishita, M., Chia, S. L., Chou, M. Y., Ding, Y. Y., Iijima, K., Jang, H. C., Kawashima, S., Kim, M., Kojima, T., Kuzuya, M., Lee, J., Lee, S. Y., Lee, Y., Peng, L. N., Wang, N. Y., Wang, Y. W., Won, C. W., Woo, J., Chen, L. K., & Arai, H. (2020). COVID-19 and older people in Asia: Asian Working Group for Sarcopenia calls to actions. *Geriatr Gerontol Int*, *20*(6), 547-558. <https://doi.org/10.1111/ggi.13939>

Longo, E., de Campos, A. C., & Schiariti, V. (2020). COVID-19 Pandemic: Is This a Good Time for Implementation of Home Programs for Children's Rehabilitation in Low- and Middle-Income Countries? *Phys Occup Ther Pediatr*, *40*(4), 361-364. <https://doi.org/10.1080/01942638.2020.1759947>

Lund, E. M. (2020). Interpersonal violence against people with disabilities: Additional concerns and considerations in the COVID-19 pandemic. *Rehabil Psychol*, *65*(3), 199-205. <https://doi.org/10.1037/rep0000347>

Lund, E. M., & Ayers, K. B. (2020). Raising awareness of disabled lives and health care rationing during the COVID-19 pandemic. *Psychol Trauma*, *12*(S1), S210-s211. <https://doi.org/10.1037/tra0000673>

Manto, M., Dupre, N., Hadjivassiliou, M., Louis, E. D., Mitoma, H., Molinari, M., Shaikh, A. G., Soong, B. W., Strupp, M., Van Overwalle, F., & Schmahmann, J. D. (2020). Medical and Paramedical Care of Patients With Cerebellar Ataxia During the COVID-19 Outbreak: Seven Practical Recommendations of the COVID 19 Cerebellum Task Force. *Front Neurol*, *11*, 516. <https://doi.org/10.3389/fneur.2020.00516>

Masonbrink, A. R., & Hurley, E. (2020). Advocating for Children During the COVID-19 School Closures. *Pediatrics*, *146*(3), 1-4. <https://doi.org/10.1542/peds.2020-1440>

McGonigal, M. (2020). Providing Quality Care to the Intellectually Disadvantaged Patient Population During the COVID-19 Pandemic. *Crit Care Nurs Q*, *43*(4), 480-483. <https://doi.org/10.1097/cnq.0000000000000331>

McKinney, E. L., McKinney, V., & Swartz, L. (2020). COVID-19, disability and the context of healthcare triage in South Africa: Notes in a time of pandemic. *African Journal of Disability*, *9*, Article a766. <https://doi.org/10.4102/ajod.v9i0.766>

Melamed, O. C., Hahn, M. K., Agarwal, S. M., Taylor, V. H., Mulsant, B. H., & Selby, P. (2020). Physical health among people with serious mental illness in the face of COVID-19: Concerns and mitigation strategies. *Gen Hosp Psychiatry*, *66*, 30-33. <https://doi.org/10.1016/j.genhosppsych.2020.06.013>

Mello, M. M., Persad, G., & White, D. B. (2020). Respecting Disability Rights — Toward Improved Crisis Standards of Care. *New England Journal of Medicine*, *383*(5), e26. <https://doi.org/10.1056/NEJMp2011997>

Mesa Vieira, C., Franco, O. H., Gómez Restrepo, C., & Abel, T. (2020). COVID-19: The forgotten priorities of the pandemic. *Maturitas*, *136*, 38-41. <https://doi.org/10.1016/j.maturitas.2020.04.004>

Middleton, A., Simpson, K. N., Bettger, J. P., & Bowden, M. G. (2020). COVID-19 Pandemic and Beyond: Considerations and Costs of Telehealth Exercise Programs for Older Adults With Functional Impairments Living at Home-Lessons Learned From a Pilot Case Study. *Phys Ther*, *100*(8), 1278-1288. <https://doi.org/10.1093/ptj/pzaa089>

Miele, G., Straccia, G., Moccia, M., Leocani, L., Tedeschi, G., Bonavita, S., & Lavorgna, L. (2020). Telemedicine in Parkinson's Disease: How to Ensure Patient Needs and Continuity of Care at the Time of COVID-19 Pandemic. *Telemed J E Health*. <https://doi.org/10.1089/tmj.2020.0184>

Mills, W. R., Sender, S., Lichtefeld, J., Romano, N., Reynolds, K., Price, M., Phipps, J., White, L., Howard, S., Poltavski, D., & Barnes, R. (2020). Supporting individuals with intellectual and developmental disability during the first 100 days of the COVID-19 outbreak in the USA. *J Intellect Disabil Res*, *64*(7), 489-496. <https://doi.org/10.1111/jir.12740>

Mukherjee, D. (2020). Experiencing Community in a Covid Surge. *Hastings Cent Rep*, *50*(3), 10-11. <https://doi.org/10.1002/hast.1109>

Muruganandam, P., Neelamegam, S., Menon, V., Alexander, J., & Chaturvedi, S. K. (2020). COVID-19 and Severe Mental Illness: Impact on patients and its relation with their awareness about COVID-19. *Psychiatry Res*, *291*, 113265. <https://doi.org/10.1016/j.psychres.2020.113265>

Palipana, D. (2020). COVID-19 and spinal cord injuries: The viewpoint from an emergency department resident with quadriplegia [Article]. *EMA - Emergency Medicine Australasia*, *32*(4), 692-693. <https://doi.org/10.1111/1742-6723.13525>

Pineda, V. S., & Corburn, J. (2020). Disability, Urban Health Equity, and the Coronavirus Pandemic: Promoting Cities for All. *J Urban Health*, *97*(3), 336-341. <https://doi.org/10.1007/s11524-020-00437-7>

Rao, S. S., Loeb, A. E., Amin, R. M., Golladay, G. J., Levin, A. S., & Thakkar, S. C. (2020). Establishing Telemedicine in an Academic Total Joint Arthroplasty Practice: Needs and Opportunities Highlighted by the COVID-19 Pandemic. *Arthroplast Today*, *6*(3), 617-622. <https://doi.org/10.1016/j.artd.2020.04.014>

Sabatello, M., Burke, T. B., McDonald, K. E., & Appelbaum, P. S. (2020). Disability, Ethics, and Health Care in the COVID-19 Pandemic. *Am J Public Health*, *110*(10), 1523-1527. <https://doi.org/10.2105/ajph.2020.305837>

Sabatello, M., Landes, S. D., & McDonald, K. E. (2020). People With Disabilities in COVID-19: Fixing Our Priorities. *American Journal of Bioethics*, *20*(7), 187-190. <https://doi.org/10.1080/15265161.2020.1779396>

Sakellariou, D., Malfitano, A. P. S., & Rotarou, E. S. (2020). Disability inclusiveness of government responses to COVID-19 in South America: a framework analysis study. *International Journal for Equity in Health*, *19*(1), Article 131. <https://doi.org/10.1186/s12939-020-01244-x>

Scully, J. L. (2020). Disability, Disablism, and COVID-19 Pandemic Triage. *J Bioeth Inq*, 1-5. <https://doi.org/10.1007/s11673-020-10005-y>

Senjam, S. S. (2020). Impact of COVID-19 pandemic on people living with visual disability. *Indian J Ophthalmol*, *68*(7), 1367-1370. <https://doi.org/10.4103/ijo.IJO_1513_20>

Sholas, M. G. (2020). The actual and potential impact of the novel 2019 coronavirus on pediatric rehabilitation: A commentary and review of its effects and potential disparate influence on Black, Latinx and Native American marginalized populations in the United States. *J Pediatr Rehabil Med*. <https://doi.org/10.3233/prm-200722>

Singh, S. (2020). Disability ethics in the coronavirus crisis. *J Family Med Prim Care*, *9*(5), 2167-2171. <https://doi.org/10.4103/jfmpc.jfmpc_588_20>

Solomon, M. Z., Wynia, M. K., & Gostin, L. O. (2020). Covid-19 Crisis Triage — Optimizing Health Outcomes and Disability Rights. *New England Journal of Medicine*, *383*(5), e27. <https://doi.org/10.1056/NEJMp2008300>

Tan, L. F., & Seetharaman, S. (2020). Preventing the Spread of COVID-19 to Nursing Homes: Experience from a Singapore Geriatric Centre. *J Am Geriatr Soc*, *68*(5), 942. <https://doi.org/10.1111/jgs.16447>

Tohidast, S. A., Mansuri, B., Bagheri, R., & Azimi, H. (2020). Provision of speech-language pathology services for the treatment of speech and language disorders in children during the COVID-19 pandemic: Problems, concerns, and solutions [Article]. *International Journal of Pediatric Otorhinolaryngology*, *138*, Article 110262. <https://doi.org/10.1016/j.ijporl.2020.110262>

Toseeb, U., Asbury, K., Code, A., Fox, L., & Deniz, E. (2020). Supporting Families with Children with Special Educational Needs and Disabilities During COVID-19. *PsyArxiv*. <https://doi.org/https://doi.org/10.31234/osf.io/tm69k>

Turk, M. A., Landes, S. D., Formica, M. K., & Goss, K. D. (2020). Intellectual and developmental disability and COVID-19 case-fatality trends: TriNetX analysis. *Disabil Health J*, *13*(3), 100942. <https://doi.org/10.1016/j.dhjo.2020.100942>

Turk, M. A., & McDermott, S. (2020). The COVID-19 pandemic and people with disability [Editorial]. *Disabil Health J*, *13*(3), Article 100944. <https://doi.org/10.1016/j.dhjo.2020.100944>

Waldman, H. B., Rader, R., Keller, S. M., & Perlman, S. P. (2020). Who's Next? *Exceptional Parent*, *50*(5), 16-18. <http://search.ebscohost.com/login.aspx?direct=true&db=cin20&AN=143154671&site=ehost-live&scope=site>

Waldman, H. B., Rader, R., & Perlman, S. P. (2020). What Are We Learning? *Exceptional Parent*, *50*(6), 17-19. <http://search.ebscohost.com/login.aspx?direct=true&db=cin20&AN=143897147&site=ehost-live&scope=site>

Woodall, T., Ramage, M., LaBruyere, J. T., McLean, W., & Tak, C. R. (2020). Telemedicine Services During COVID-19: Considerations for Medically Underserved Populations. *J Rural Health*. <https://doi.org/10.1111/jrh.12466>

Wright, C., Steinway, C., & Jan, S. (2020). The Crisis Close at Hand: How COVID-19 Challenges Long-Term Care Planning for Adults with Intellectual Disability. *Health Equity*, *4*(1), 247-248. <https://doi.org/10.1089/heq.2020.0020>

Yap, J., Chaudhry, V., Jha, C. K., Mani, S., & Mitra, S. (2020). Are responses to the pandemic inclusive? A rapid virtual audit of COVID-19 press briefings in LMICs. *World Dev*, *136*, 105122. <https://doi.org/10.1016/j.worlddev.2020.105122>

Zaagsma, M., Volkers, K. M., Swart, E. A. K., Schippers, A. P., & Van Hove, G. The use of online support by people with intellectual disabilities living independently during COVID-19. *Journal of Intellectual Disability Research*. <https://doi.org/10.1111/jir.12770>
