# Supplementary material for: PREparedness, REsponse and SySTemic transformation (PRE-RE-SyST): a model for disability-inclusive pandemic responses and systemic disparities reduction derived from a scoping review and thematic analysis
Source: Int J Equity Health. 2021 Sep 14;20:204. doi: 10.1186/s12939-021-01526-y (PMC8438560; doi:10.1186/s12939-021-01526-y)
Supplement: Supplementary file 1 — Additional file 1. [file 12939_2021_1526_MOESM1_ESM.zip › Web-appendix 3 - Characteristics of papers included.docx]

| **Characteristics** | **#** | **%** | **Citations** |
| --- | --- | --- | --- |
| **PUBLICATIONS TYPE AND SOURCE** | | | |
| Perspective papers (e.g., viewpoints, commentaries, essays, ethics/advocacy) | 45 | 54% | ^1-45^ |
| Narrative summary/review (non-systematic) | 8 | 10% | ^46-53^ |
| Editorial or Letter to the editor | 6 | 7% | ^54-59^ |
| Framework or Experts recommendations | 3 | 4% | ^60-62^ |
| **Non-empirical (peer-reviewed): SUB-TOTAL** | **62** | **74**% | **-** |
| Cross-sectional surveys | 4 | 5% | ^63-66^ |
| Institutional case report | 4 | 5% | ^67-70^ |
| Pilot study | 2 | 2% | ^71 72^ |
| Systematic analysis of electronic medical/ administrative records | 2 | 1% | ^73 74^ |
| Ecological study | 1 | 1% | ^75^ |
| Analysis of COVID-19 press conferences | 1 | 1% | ^76^ |
| Analysis of webpages on accessibility compliance | 1 | 1% | ^77^ |
| Documentary research and framework analysis | 1 | 1% | ^78^ |
| Analysis of contacts to online support services | 1 | 1% | ^79^ |
| Survey research, with qualitative analysis | 1 | 1% | ^80^ |
| Country case report | 1 | 1% | ^81^ |
| **Empirical studies (peer-reviewed): SUB-TOTAL** | **19** | **23**% | **-** |
| Survey research | 1 | 1% | ^82^ |
| Observational multi-center study | 1 | 1% | ^83^ |
| Comparative cross-sectional survey (control group) | 1 | 1% | ^84^ |
| **Preprint studies: SUB-TOTAL** | **3** | **4**% | **-** |
| **GEOGRAPHICAL FOCUS** | | | |
| No geographical focus (e.g., applicable across locations) | 52 | 62% | ^1-8 10-14 16-19 21-25 27 28 30 31 33 35-38 40 42-45 47 49 50 52-59 61 62 73 75 77^ |
| United States of America (USA) | 10 | 12% | ^15 26 29 48 51 60 69-71 74^ |
| United Kingdom (UK) | 7 | 8% | ^34 41 64 80 82-84^ |
| Low- and Middle-Income countries (LMICs) | 4 | 5% | ^9 20 39 76^ |
| Spain | 2 | 2% | ^66 67^ |
| Italy | 2 | 2% | ^63 72^ |
| Netherlands | 1 | 1% | ^79^ |
| Singapore | 1 | 1% | ^68^ |
| South Korea | 1 | 1% | ^81^ |
| South Africa | 1 | 1% | ^32^ |
| India | 1 | 1% | ^65^ |
| Asia | 1 | 1% | ^46^ |
| Latin America | 1 | 1% | ^78^ |
| **HEALTH CONDITIONS** | | | |
| People with Disabilities (PwD) Overall | 33 | 39% | ^2-6 8 10 14 20-24 27 28 32-34 38-44 48 56 59 64 76-78 81^ |
| Adults with cognitive impairment (e.g., dementia) or intellectual/developmental disabilities | 17 | 20% | ^12 16 26 30 35 37 45 54 58 61 66 67 69 73-75 79^ |
| Children/youth with disabilities (and their families) | 10 | 12% | ^9 13 15 29 36 50 55 70 80 82^ |
| Older adults experiencing disabilities | 9 | 11% | ^1 7 19 46 49 53 57 60 71^ |
| Severe Mental Illness | 2 | 2% | ^31 47^ |
| Spinal Cord Injury | 2 | 2% | ^68 83^ |
| PwD living in residential or long-term facilities | 2 | 2% | ^11 65^ |
| Visual impairments | 1 | 1% | ^25^ |
| Autism Spectrum Disorder | 1 | 1% | ^17^ |
| Cerebral Palsy | 1 | 1% | ^18^ |
| Cerebellar Ataxia | 1 | 1% | ^62^ |
| Amyotrophic Lateral Sclerosis | 1 | 1% | ^72^ |
| Parkinson’s | 1 | 1% | ^52^ |
| Multiple sclerosis | 1 | 1% | ^63^ |
| People requiring joint surgery | 1 | 1% | ^51^ |
| Chronic pain | 1 | 1% | ^84^ |

**References**

1. Chase J. Caring for Frail Older Adults During COVID-19: Integrating Public Health Ethics into Clinical Practice. *Journal of the American Geriatrics Society* 2020;68(8):1666-70. doi: 10.1111/jgs.16666 [published Online First: 2020/06/20]

2. Chen B, McNamara DM. Disability Discrimination, Medical Rationing and COVID-19. *Asian bioethics review* 2020:1-8. doi: 10.1007/s41649-020-00147-x [published Online First: 2020/09/10]

3. Singh S. Disability ethics in the coronavirus crisis. *Journal of family medicine and primary care* 2020;9(5):2167-71. doi: 10.4103/jfmpc.jfmpc_588_20 [published Online First: 2020/08/06]

4. Scully JL. Disability, Disablism, and COVID-19 Pandemic Triage. *Journal of bioethical inquiry* 2020:1-5. doi: 10.1007/s11673-020-10005-y [published Online First: 2020/08/26]

5. Sabatello M, Burke TB, McDonald KE, et al. Disability, Ethics, and Health Care in the COVID-19 Pandemic. *American journal of public health* 2020;110(10):1523-27. doi: 10.2105/ajph.2020.305837 [published Online First: 2020/08/21]

6. Sabatello M, Landes SD, McDonald KE. People With Disabilities in COVID-19: Fixing Our Priorities. *American Journal of Bioethics* 2020;20(7):187-90. doi: 10.1080/15265161.2020.1779396

7. Farrell TW, Ferrante LE, Brown T, et al. AGS Position Statement: Resource Allocation Strategies and Age-Related Considerations in the COVID-19 Era and Beyond. *Journal of the American Geriatrics Society* 2020;68(6):1136-42. doi: 10.1111/jgs.16537 [published Online First: 2020/05/07]

8. Lund EM, Ayers KB. Raising awareness of disabled lives and health care rationing during the COVID-19 pandemic. *Psychological trauma : theory, research, practice and policy* 2020;12(S1):S210-s11. doi: 10.1037/tra0000673 [published Online First: 2020/05/29]

9. Longo E, de Campos AC, Schiariti V. COVID-19 Pandemic: Is This a Good Time for Implementation of Home Programs for Children's Rehabilitation in Low- and Middle-Income Countries? *Physical & occupational therapy in pediatrics* 2020;40(4):361-64. doi: 10.1080/01942638.2020.1759947 [published Online First: 2020/05/16]

10. Fitzgerald H, Stride A, Drury S. COVID-19, lockdown and (disability) sport. *Managing Sport and Leisure* 2020:1-8. doi: 10.1080/23750472.2020.1776950

11. Melamed OC, Hahn MK, Agarwal SM, et al. Physical health among people with serious mental illness in the face of COVID-19: Concerns and mitigation strategies. *General hospital psychiatry* 2020;66:30-33. doi: 10.1016/j.genhosppsych.2020.06.013 [published Online First: 2020/07/10]

12. Woodall T, Ramage M, LaBruyere JT, et al. Telemedicine Services During COVID-19: Considerations for Medically Underserved Populations. *The Journal of rural health : official journal of the American Rural Health Association and the National Rural Health Care Association* 2020 doi: 10.1111/jrh.12466 [published Online First: 2020/07/03]

13. Aishworiya R, Kang YQ. Including Children with Developmental Disabilities in the Equation During this COVID-19 Pandemic. *Journal of autism and developmental disorders* 2020:1-4. doi: 10.1007/s10803-020-04670-6 [published Online First: 2020/08/21]

14. Annaswamy TM, Verduzco-Gutierrez M, Frieden L. Telemedicine barriers and challenges for persons with disabilities: Covid-19 and beyond. *Disability and health journal* 2020:100973. doi: 10.1016/j.dhjo.2020.100973 [published Online First: 2020/07/25]

15. Sholas MG. The actual and potential impact of the novel 2019 coronavirus on pediatric rehabilitation: A commentary and review of its effects and potential disparate influence on Black, Latinx and Native American marginalized populations in the United States. *Journal of pediatric rehabilitation medicine* 2020 doi: 10.3233/prm-200722 [published Online First: 2020/07/28]

16. Iaboni A, Cockburn A, Marcil M, et al. Achieving Safe, Effective, and Compassionate Quarantine or Isolation of Older Adults With Dementia in Nursing Homes. *The American journal of geriatric psychiatry : official journal of the American Association for Geriatric Psychiatry* 2020;28(8):835-38. doi: 10.1016/j.jagp.2020.04.025 [published Online First: 2020/05/21]

17. Cox DJ, Plavnick JB, Brodhead MT. A Proposed Process for Risk Mitigation During the COVID-19 Pandemic. *Behavior analysis in practice* 2020;13(2):1-7. doi: 10.1007/s40617-020-00430-1 [published Online First: 2020/04/25]

18. Ben-Pazi H, Beni-Adani L, Lamdan R. Accelerating Telemedicine for Cerebral Palsy During the COVID-19 Pandemic and Beyond. *Frontiers in neurology* 2020;11:746. doi: 10.3389/fneur.2020.00746 [published Online First: 2020/07/17]

19. Eghtesadi M. Breaking Social Isolation Amidst COVID-19: A Viewpoint on Improving Access to Technology in Long-Term Care Facilities. *Journal of the American Geriatrics Society* 2020;68(5):949-50. doi: 10.1111/jgs.16478 [published Online First: 2020/04/12]

20. Mesa Vieira C, Franco OH, Gómez Restrepo C, et al. COVID-19: The forgotten priorities of the pandemic. *Maturitas* 2020;136:38-41. doi: 10.1016/j.maturitas.2020.04.004 [published Online First: 2020/05/11]

21. Guidry-Grimes L, Savin K, Stramondo JA, et al. Disability Rights as a Necessary Framework for Crisis Standards of Care and the Future of Health Care. *The Hastings Center report* 2020;50(3):28-32. doi: 10.1002/hast.1128 [published Online First: 2020/07/01]

22. Goggin G, Ellis K. Disability, communication, and life itself in the COVID-19 pandemic. *Health Sociology Review* 2020;29(2):168-76. doi: 10.1080/14461242.2020.1784020

23. Pineda VS, Corburn J. Disability, Urban Health Equity, and the Coronavirus Pandemic: Promoting Cities for All. *Journal of urban health : bulletin of the New York Academy of Medicine* 2020;97(3):336-41. doi: 10.1007/s11524-020-00437-7 [published Online First: 2020/04/25]

24. Mukherjee D. Experiencing Community in a Covid Surge. *The Hastings Center report* 2020;50(3):10-11. doi: 10.1002/hast.1109 [published Online First: 2020/05/06]

25. Senjam SS. Impact of COVID-19 pandemic on people living with visual disability. *Indian journal of ophthalmology* 2020;68(7):1367-70. doi: 10.4103/ijo.IJO_1513_20 [published Online First: 2020/06/27]

26. Wright C, Steinway C, Jan S. The Crisis Close at Hand: How COVID-19 Challenges Long-Term Care Planning for Adults with Intellectual Disability. *Health equity* 2020;4(1):247-48. doi: 10.1089/heq.2020.0020 [published Online First: 2020/06/27]

27. Waldman HB, Rader R, Perlman SP. What Are We Learning? *Exceptional Parent* 2020;50(6):17-19.

28. Waldman HB, Rader R, Keller SM, et al. Who's Next? *Exceptional Parent* 2020;50(5):16-18.

29. Masonbrink AR, Hurley E. Advocating for Children During the COVID-19 School Closures. *Pediatrics* 2020;146(3):1-4. doi: 10.1542/peds.2020-1440

30. Brown EE, Kumar S, Rajji TK, et al. Anticipating and Mitigating the Impact of the COVID-19 Pandemic on Alzheimer's Disease and Related Dementias. *Am J Geriatr Psychiatry* 2020;28(7):712-21. doi: 10.1016/j.jagp.2020.04.010

31. Palipana D. COVID-19 and spinal cord injuries: The viewpoint from an emergency department resident with quadriplegia. *EMA Emerg Med Australas* 2020;32(4):692-93. doi: 10.1111/1742-6723.13525

32. McKinney EL, McKinney V, Swartz L. COVID-19, disability and the context of healthcare triage in South Africa: Notes in a time of pandemic. *African Journal of Disability* 2020;9 doi: 10.4102/ajod.v9i0.766

33. Lund EM. Interpersonal violence against people with disabilities: Additional concerns and considerations in the COVID-19 pandemic. *Rehabilitation psychology* 2020;65(3):199-205. doi: 10.1037/rep0000347 [published Online First: 2020/08/18]

34. Eskytė I, Lawson A, Orchard M, et al. Out on the Streets - Crisis, Opportunity and Disabled People in the Era of Covid-19: Reflections from the UK. *Alter* 2020 doi: 10.1016/j.alter.2020.07.004 [published Online First: 2020/08/25]

35. McGonigal M. Providing Quality Care to the Intellectually Disadvantaged Patient Population During the COVID-19 Pandemic. *Critical care nursing quarterly* 2020;43(4):480-83. doi: 10.1097/cnq.0000000000000331 [published Online First: 2020/08/25]

36. Tohidast SA, Mansuri B, Bagheri R, et al. Provision of speech-language pathology services for the treatment of speech and language disorders in children during the COVID-19 pandemic: Problems, concerns, and solutions. *Int J Pediatr Otorhinolaryngol* 2020;138 doi: 10.1016/j.ijporl.2020.110262

37. Devita M, Bordignon A, Sergi G, et al. The psychological and cognitive impact of Covid-19 on individuals with neurocognitive impairments: research topics and remote intervention proposals. *Aging Clinical and Experimental Research* doi: 10.1007/s40520-020-01637-6

38. Andrews EE, Ayers KB, Brown KS, et al. No body is expendable: Medical rationing and disability justice during the COVID-19 pandemic. *The American psychologist* 2020 doi: 10.1037/amp0000709 [published Online First: 2020/07/24]

39. Banks LM, Davey C, Shakespeare T, et al. Disability-inclusive responses to COVID-19: Lessons learnt from research on social protection in low- and middle-income countries. *World development* 2021;137:105178. doi: 10.1016/j.worlddev.2020.105178 [published Online First: 2020/09/10]

40. Jesus TS, Landry MD, Jacobs K. A 'new normal' following COVID-19 and the economic crisis: Using systems thinking to identify challenges and opportunities in disability, telework, and rehabilitation. *Work (Reading, Mass)* 2020;67(1):37-46. doi: 10.3233/wor-203250 [published Online First: 2020/09/22]

41. Kuper H, Banks LM, Bright T, et al. Disability-inclusive COVID-19 response: What it is, why it is important and what we can learn from the United Kingdom's response. *Wellcome open research* 2020;5:79. doi: 10.12688/wellcomeopenres.15833.1 [published Online First: 2020/06/06]

42. Boyle CA, Fox MH, Havercamp SM, et al. The public health response to the COVID-19 pandemic for people with disabilities. *Disability and health journal* 2020;13(3):100943. doi: 10.1016/j.dhjo.2020.100943 [published Online First: 2020/06/06]

43. Mello MM, Persad G, White DB. Respecting Disability Rights — Toward Improved Crisis Standards of Care. *New England Journal of Medicine* 2020;383(5):e26. doi: 10.1056/NEJMp2011997

44. Solomon MZ, Wynia MK, Gostin LO. Covid-19 Crisis Triage — Optimizing Health Outcomes and Disability Rights. *New England Journal of Medicine* 2020;383(5):e27. doi: 10.1056/NEJMp2008300

45. Courtenay K, Perera B. COVID-19 and people with intellectual disability: impacts of a pandemic. *Irish journal of psychological medicine* 2020;37(3):231-36. doi: 10.1017/ipm.2020.45 [published Online First: 2020/05/15]

46. Lim WS, Liang CK, Assantachai P, et al. COVID-19 and older people in Asia: Asian Working Group for Sarcopenia calls to actions. *Geriatrics & gerontology international* 2020;20(6):547-58. doi: 10.1111/ggi.13939 [published Online First: 2020/05/05]

47. Korupolu R, Stampas A, Gibbons C, et al. COVID-19: Screening and triage challenges in people with disability due to Spinal Cord Injury. *Spinal cord series and cases* 2020;6(1):35. doi: 10.1038/s41394-020-0284-7 [published Online First: 2020/05/13]

48. Blewett LA, Hest R. Emergency Flexibility for States to Increase and Maintain Medicaid Eligibility for LTSS under COVID-19. *Journal of aging & social policy* 2020;32(4-5):343-49. doi: 10.1080/08959420.2020.1774312 [published Online First: 2020/06/02]

49. Banskota S, Healy M, Goldberg EM. 15 Smartphone Apps for Older Adults to Use While in Isolation During the COVID-19 Pandemic. *The western journal of emergency medicine* 2020;21(3):514-25. doi: 10.5811/westjem.2020.4.47372 [published Online First: 2020/04/18]

50. Fegert JM, Vitiello B, Plener PL, et al. Challenges and burden of the Coronavirus 2019 (COVID-19) pandemic for child and adolescent mental health: a narrative review to highlight clinical and research needs in the acute phase and the long return to normality. *Child and adolescent psychiatry and mental health* 2020;14:20. doi: 10.1186/s13034-020-00329-3 [published Online First: 2020/05/19]

51. Rao SS, Loeb AE, Amin RM, et al. Establishing Telemedicine in an Academic Total Joint Arthroplasty Practice: Needs and Opportunities Highlighted by the COVID-19 Pandemic. *Arthroplasty today* 2020;6(3):617-22. doi: 10.1016/j.artd.2020.04.014 [published Online First: 2020/04/25]

52. Miele G, Straccia G, Moccia M, et al. Telemedicine in Parkinson's Disease: How to Ensure Patient Needs and Continuity of Care at the Time of COVID-19 Pandemic. *Telemedicine journal and e-health : the official journal of the American Telemedicine Association* 2020 doi: 10.1089/tmj.2020.0184 [published Online First: 2020/07/16]

53. D'Cruz M, Banerjee D. 'An invisible human rights crisis': The marginalization of older adults during the COVID-19 pandemic - An advocacy review. *Psychiatry research* 2020;292:113369. doi: 10.1016/j.psychres.2020.113369 [published Online First: 2020/08/17]

54. Lee OE, Davis B. Adapting 'Sunshine,' A Socially Assistive Chat Robot for Older Adults with Cognitive Impairment: A Pilot Study. *Journal of gerontological social work* 2020:1-3. doi: 10.1080/01634372.2020.1789256 [published Online First: 2020/07/09]

55. Fazzi E, Galli J. New clinical needs and strategies for care in children with neurodisability during COVID-19. *Developmental medicine and child neurology* 2020;62(7):879-80. doi: 10.1111/dmcn.14557 [published Online First: 2020/05/03]

56. Armitage R, Nellums LB. The COVID-19 response must be disability inclusive. *The Lancet Public health* 2020;5(5):e257. doi: 10.1016/s2468-2667(20)30076-1 [published Online First: 2020/04/01]

57. Frost R, Nimmons D, Davies N. Using Remote Interventions in Promoting the Health of Frail Older Persons Following the COVID-19 Lockdown: Challenges and Solutions. *Journal of the American Medical Directors Association* 2020;21(7):992-93. doi: 10.1016/j.jamda.2020.05.038 [published Online First: 2020/06/20]

58. Cheung G, Peri K. Challenges to dementia care during COVID-19: Innovations in remote delivery of group Cognitive Stimulation Therapy. *Aging & mental health* 2020:1-3. doi: 10.1080/13607863.2020.1789945 [published Online First: 2020/07/08]

59. Turk MA, McDermott S. The COVID-19 pandemic and people with disability. *Disability and health journal* 2020;13(3) doi: 10.1016/j.dhjo.2020.100944

60. Hoffman GJ, Webster NJ, Bynum JPW. A Framework for Aging-Friendly Services and Supports in the Age of COVID-19. *Journal of aging & social policy* 2020;32(4-5):450-59. doi: 10.1080/08959420.2020.1771239 [published Online First: 2020/05/23]

61. Alexander R, Ravi A, Barclay H, et al. Guidance for the Treatment and Management of COVID-19 Among People with Intellectual Disabilities. *Journal of policy and practice in intellectual disabilities* 2020 doi: 10.1111/jppi.12352 [published Online First: 2020/08/25]

62. Manto M, Dupre N, Hadjivassiliou M, et al. Medical and Paramedical Care of Patients With Cerebellar Ataxia During the COVID-19 Outbreak: Seven Practical Recommendations of the COVID 19 Cerebellum Task Force. *Frontiers in neurology* 2020;11:516. doi: 10.3389/fneur.2020.00516 [published Online First: 2020/06/24]

63. Landi D, Ponzano M, Nicoletti CG, et al. Adherence to social distancing and use of personal protective equipment and the risk of SARS-CoV-2 infection in a cohort of patients with multiple sclerosis. *Multiple sclerosis and related disorders* 2020;45:102359. doi: 10.1016/j.msard.2020.102359 [published Online First: 2020/07/15]

64. Iob E, Steptoe A, Fancourt D. Abuse, self-harm and suicidal ideation in the UK during the COVID-19 pandemic. *The British journal of psychiatry : the journal of mental science* 2020;217(4):543-46. doi: 10.1192/bjp.2020.130 [published Online First: 2020/07/14]

65. Muruganandam P, Neelamegam S, Menon V, et al. COVID-19 and Severe Mental Illness: Impact on patients and its relation with their awareness about COVID-19. *Psychiatry research* 2020;291:113265. doi: 10.1016/j.psychres.2020.113265 [published Online First: 2020/08/09]

66. Goodman-Casanova JM, Dura-Perez E, Guzman-Parra J, et al. Telehealth Home Support During COVID-19 Confinement for Community-Dwelling Older Adults With Mild Cognitive Impairment or Mild Dementia: Survey Study. *Journal of medical Internet research* 2020;22(5):e19434. doi: 10.2196/19434 [published Online First: 2020/05/14]

67. Benaque A, Gurruchaga MJ, Abdelnour C, et al. Dementia Care in Times of COVID-19: Experience at Fundació ACE in Barcelona, Spain. *Journal of Alzheimer's disease : JAD* 2020;76(1):33-40. doi: 10.3233/jad-200547 [published Online First: 2020/06/17]

68. Tan LF, Seetharaman S. Preventing the Spread of COVID-19 to Nursing Homes: Experience from a Singapore Geriatric Centre. *Journal of the American Geriatrics Society* 2020;68(5):942. doi: 10.1111/jgs.16447 [published Online First: 2020/03/28]

69. Mills WR, Sender S, Lichtefeld J, et al. Supporting individuals with intellectual and developmental disability during the first 100 days of the COVID-19 outbreak in the USA. *Journal of intellectual disability research : JIDR* 2020;64(7):489-96. doi: 10.1111/jir.12740 [published Online First: 2020/06/04]

70. Frederick JK, Raabe GR, Rogers VR, et al. Advocacy, collaboration, and intervention: A model of distance special education support services amid covid-19. *Behavior analysis in practice* 2020 doi: 10.1007/s40617-020-00476-1

71. Middleton A, Simpson KN, Bettger JP, et al. COVID-19 Pandemic and Beyond: Considerations and Costs of Telehealth Exercise Programs for Older Adults With Functional Impairments Living at Home-Lessons Learned From a Pilot Case Study. *Physical therapy* 2020;100(8):1278-88. doi: 10.1093/ptj/pzaa089 [published Online First: 2020/05/07]

72. Capozzo R, Zoccolella S, Musio M, et al. Telemedicine is a useful tool to deliver care to patients with Amyotrophic Lateral Sclerosis during COVID-19 pandemic: results from Southern Italy. *Amyotrophic lateral sclerosis & frontotemporal degeneration* 2020:1-7. doi: 10.1080/21678421.2020.1773502 [published Online First: 2020/06/13]

73. Turk MA, Landes SD, Formica MK, et al. Intellectual and developmental disability and COVID-19 case-fatality trends: TriNetX analysis. *Disability and health journal* 2020;13(3):100942. doi: 10.1016/j.dhjo.2020.100942 [published Online First: 2020/06/01]

74. Landes SD, Turk MA, Formica MK, et al. COVID-19 outcomes among people with intellectual and developmental disability living in residential group homes in New York State. *Disability and health journal* 2020;13(4):100969. doi: 10.1016/j.dhjo.2020.100969 [published Online First: 2020/07/01]

75. Azarpazhooh MR, Amiri A, Morovatdar N, et al. Correlations between COVID-19 and burden of dementia: An ecological study and review of literature. *Journal of the neurological sciences* 2020;416:117013. doi: 10.1016/j.jns.2020.117013 [published Online First: 2020/07/14]

76. Yap J, Chaudhry V, Jha CK, et al. Are responses to the pandemic inclusive? A rapid virtual audit of COVID-19 press briefings in LMICs. *World development* 2020;136:105122. doi: 10.1016/j.worlddev.2020.105122 [published Online First: 2020/08/25]

77. Fernández-Díaz E, Iglesias-Sánchez PP, Jambrino-Maldonado C. Exploring WHO Communication during the COVID 19 Pandemic through the WHO Website Based on W3C Guidelines: Accessible for All? *International journal of environmental research and public health* 2020;17(16) doi: 10.3390/ijerph17165663 [published Online First: 2020/08/09]

78. Sakellariou D, Malfitano APS, Rotarou ES. Disability inclusiveness of government responses to COVID-19 in South America: a framework analysis study. *International Journal for Equity in Health* 2020;19(1) doi: 10.1186/s12939-020-01244-x

79. Zaagsma M, Volkers KM, Swart EAK, et al. The use of online support by people with intellectual disabilities living independently during COVID-19. *Journal of Intellectual Disability Research* doi: 10.1111/jir.12770

80. Asbury K, Fox L, Deniz E, et al. How is COVID-19 Affecting the Mental Health of Children with Special Educational Needs and Disabilities and Their Families? *Journal of autism and developmental disorders* 2020:1-9. doi: 10.1007/s10803-020-04577-2 [published Online First: 2020/08/02]

81. Lee S, Kim J. A country report: Impact of covid-19 and inequity of health on south korea’s disabled community during a pandemic. *Disability & Society* 2020 doi: 10.1080/09687599.2020.1809352

82. Toseeb U, Asbury, K., Code, A., Fox, L., & Deniz, E. Supporting Families with Children with Special Educational Needs and Disabilities During COVID-19. *PsyArxiv* 2020 doi: <https://doi.org/10.31234/osf.io/tm69k>

83. Balestrini S, Koepp MJ, Gandhi S, et al. Clinical outcomes of SARS-CoV-2 pandemic in long-term care facilities for people with epilepsy: observational study. *medRxiv* 2020:2020.06.10.20123281. doi: 10.1101/2020.06.10.20123281

84. Fallon N, Brown C, Twiddy H, et al. Adverse effects of COVID-19 related lockdown on pain, physical activity and psychological wellbeing in people with chronic pain. *medRxiv* 2020:2020.06.04.20122564. doi: 10.1101/2020.06.04.20122564
